# ERAD machinery controls the conditional turnover of PIN-LIKES in plants

**DOI:** 10.1126/sciadv.adx5027

**Published:** 2025-09-19

**Authors:** Seinab Noura, Jonathan Ferreira Da Silva Santos, Elena Feraru, Sebastian N. W. Hoernstein, Mugurel I. Feraru, Laura Montero-Morales, Ann-Kathrin Rößling, David Scheuring, Richard Strasser, Pitter F. Huesgen, Sascha Waidmann, Jürgen Kleine-Vehn

**Affiliations:** ^1^ Institute of Biology II, Chair of Molecular Plant Physiology (MoPP), University of Freiburg, 79104 Freiburg, Germany.; ^2^ Center for Integrative Biological Signalling Studies (CIBSS), University of Freiburg, 79104 Freiburg, Germany.; ^3^ Institute of Molecular Plant Biology (IMPB), Department of Applied Genetics and Cell Biology, University of Natural Resources and Life Sciences (BOKU), Vienna, 1190 Vienna, Austria.; ^4^ Institute of Biology II, Biochemistry and Functional Proteomics, University of Freiburg, 79104 Freiburg, Germany.; ^5^ Institute of Plant Biotechnology and Cell Biology (IPBT), Department of Applied Genetics and Cell Biology, University of Natural Resources and Life Sciences (BOKU), Vienna, 1190 Vienna, Austria.

## Abstract

Auxin is a crucial phytohormone that regulates plant development and facilitates dynamic responses to environmental changes through subcellular control mechanisms. PIN-LIKES (PILS) are auxin transport facilitators at the endoplasmic reticulum (ER) that mediate nuclear auxin abundance and signaling. Although the posttranslational regulation of PILS is important for acclimating growth responses, the molecular mechanisms involved remain largely unknown. This study demonstrates that components of the ER-associated degradation (ERAD) machinery regulate the proteasome-dependent degradation of functional PILS proteins under nonstressed conditions. We further reveal that both internal and external signals use the ERAD complex to differentially modulate the turnover rates of PILS proteins. Our findings uncover an additional physiological role of the ERAD complex in regulating PILS protein turnover. This finding uncovers the interplay between protein homeostasis at the ER and growth regulation, opening unexplored molecular avenues into how plants acclimate to internal and external cues.

## Teaser

Plant ERAD machinery governs the turnover of auxin transporters involved in developmental and stress responses.

## INTRODUCTION

The phytohormone auxin plays a central role in coordinating plant growth responses to internal and external signals. These adaptive auxin responses largely depend on the modulation of auxin transport processes, which ensure precise distribution and availability of this hormone throughout the plant. Although considerable progress has been made in understanding intercellular auxin transport, intracellular mechanisms remain less well characterized. Recent evidence suggests that subcellular control of auxin is critical for plant acclimation to environmental fluctuations (*1*–*6*).

The PIN-LIKES (PILS) family of auxin transport facilitators, localized at the endoplasmic reticulum (ER), plays a role in regulating nuclear auxin levels and thereby signaling (*1*, *3*, *7*). This regulation is thought to occur through a compartmentalization effect that limits auxin diffusion into the nucleus. PILS protein abundance is posttranslationally regulated by a variety of environmental cues, including light, temperature, and ER stress, as well as internal phytohormonal signals, such as brassinosteroids and auxin (*3*, *5*, *6*).

By integrating these signals, PILS proteins contribute to the acclimation of auxin-dependent growth (*2*, *3*, *5*, *6*). Despite their developmental significance, the mechanisms by which ER-resident proteins, including PILS, are regulated and subjected to degradation remain poorly understood.

The ER-associated degradation (ERAD) complex is essential for degrading misfolded or defective proteins, thereby maintaining ER homeostasis in plants (*8*). Beyond quality control, the ERAD complex is increasingly recognized to regulate the conditional degradation of properly folded proteins, often referred to as physiological clients (*9*–*12*). Here, we explore the role of the ERAD complex in the conditional degradation of functional PILS proteins, pinpointing a role in plant growth regulation.

## RESULTS

Canonical PIN-FORMED (PIN) auxin carriers are predominantly located at the plasma membrane and are trafficked to the vacuole for lytic degradation (*13*–*15*). Given that vacuolar cargo sorting can initiate in the ER (*16*), we initially hypothesized that PILS proteins might similarly be transported to the vacuole for degradation. Functional PILS–green fluorescent protein (GFP) fusions (*1*–*3*, *5*, *6*) predominantly remain in the ER and were unaffected by brefeldin A (BFA), a vesicle trafficking inhibitor that also disrupts vacuolar cargo delivery (*17*) (fig. S1, A and B). Moreover, treatment with Vacuolar Affecting Compound 1 (VAC1), which induces the accumulation of vacuolar cargos (*18*), did not inhibit but promoted the degradation of PILS6 (fig. S1, C and D). These findings suggest that, unlike canonical PIN proteins, PILS proteins do not rely on BFA- or VAC1-sensitive trafficking pathways for their lytic degradation.

**Fig. 1.**
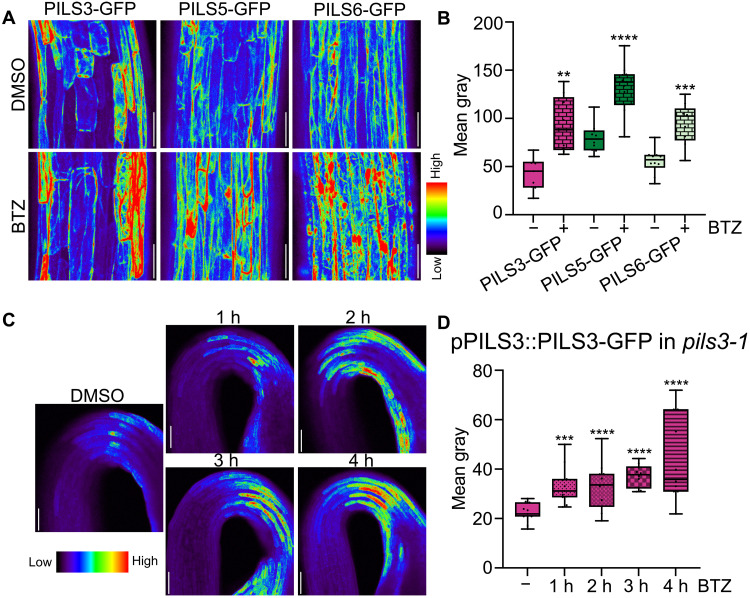
Interference with the proteasome increases PILS abundance. (**A** and **B**) Representative images (A) and quantifications (B) of GFP-PILS3, PILS5-GFP, and PILS6-GFP signal in 3-day-old dark-grown seedlings. Seedlings were grown on solid ½ MS and treated with DMSO or 40 μM BTZ in liquid ½ MS for 4 hours. Scale bars, 50 μm. *n* = 9 to 11, Student’s *t* test between DMSO and BTZ. (**C** and **D**) Representative images (C) and quantifications (D) of pPILS3::PILS3-GFP in the *pils3-1* background in 3-day-old dark-grown seedlings. Seedlings were grown on solid ½ MS and treated with DMSO or 40 μM BTZ in liquid ½ MS for 1 to 4 hours. h, hours. *n* = 10, one-way analysis of variance (ANOVA) followed by Tukey’s multiple comparisons between DMSO and BTZ. In all panels with box plots: Box limits represent the 25th percentile and 75th percentile; horizontal line represents the median. Whiskers display min. to max. values. *P* values: **P < 0.01, ***P < 0.001, and ****P < 0.0001. All experiments were repeated at least three times.

To further investigate the regulatory mechanisms governing PILS protein turnover, we assessed the role of the cytosolic 26*S* proteasome using the pharmacological inhibitor bortezomib (BTZ) (*19*). The application of the proteasome inhibitor BTZ resulted in increased fluorescence of constitutively expressed GFP-PILS3, PILS5-GFP, and PILS6-GFP in dark-grown hypocotyls compared to solvent control treatments (Fig. 1, A and B). This observation suggests a general posttranslational regulation of PILS proteins. These findings strongly indicate that the turnover of PILS proteins depends on the functionality of the 26*S* proteasome. In addition, BTZ application notably increased the abundance of pPILS3::PILS3-GFP in apical hooks (in the *pils3-1* mutant) (Fig. 1, C and D) but did not influence PILS3 expression (fig. S1E). This set of data illustrates that proteasomal degradation strongly affects the physiological levels of PILS3 proteins. Notably, the effects of proteasome inhibition on PILS3 abundance were observed within just 1 hour (Fig. 1D), suggesting a rapid and likely direct mode of action.

**Fig. 2.**
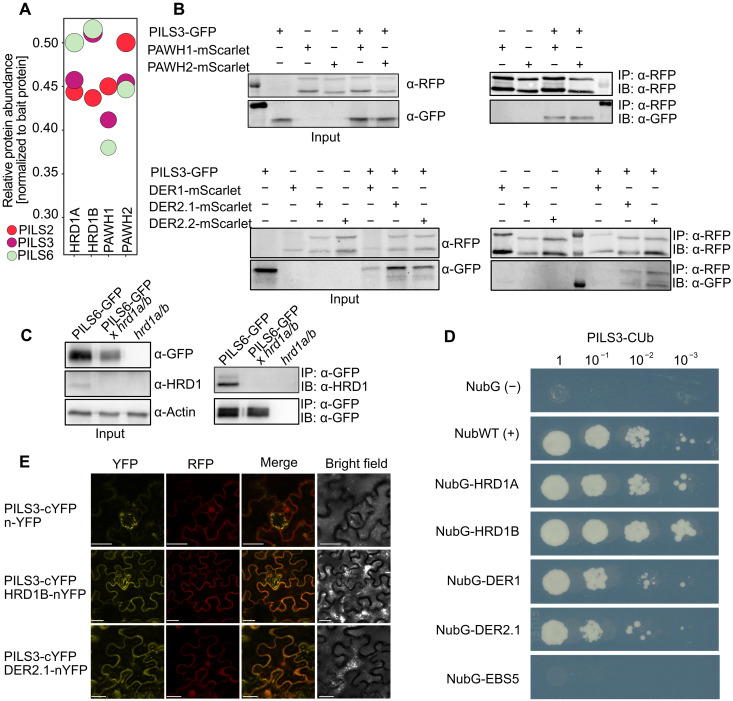
PILS proteins interact with components of the ERAD complex. (**A**) Relative protein abundance of coimmunoprecipitated proteins, using GFP-PILS2, GFP-PILS3, and PILS6-GFP as baits. HRD1A/B and PAWH1/2 were among the most abundant interactors from mass spectrometry experiments. (**B**) Co-IP of PILS3-GFP with PAWH1/2-mScarlet and DER1/2.1/2.2-mScarlet transiently expressed in *N. benthamiana*. Inputs and α-RFP or α-GFP immunoprecipitates (IPs) were separated by SDS–polyacrylamide gel electrophoresis (PAGE) and analyzed by immunoblotting (IB) with RFP or GFP antibodies. The same amount of protein was used for each IP. (**C**) Co-IP of PILS6-GFP with anti HRD1 antibody in stable *A. thaliana* transgenics. Inputs and α-GFP IPs were separated by SDS-PAGE and analyzed by IB with GFP and HRD1 antibodies. The same amount of protein was used for each IP. (**D**) Mating-based split-ubiquitin assay between PILS3 and the indicated proteins. Growth of transformed yeast colonies was detected after 5 days at room temperature. NubG was used as a negative control and NubWT as a positive control. (**E**) rBiFC in *N. benthamiana* leaves transiently transformed with constructs encoding PILS3-cYFP and HRD1B-, DER2.1-nYFP, or nYFP alone. A constitutively expressed mRFP (from the same T-DNA) was used as expression control (*30*). Scale bars, 25 μm. The proteomic screen was performed twice (A), whereas all other experiments were repeated at least three times.

To molecularly elucidate the proteasome-dependent turnover of PILS proteins, we subsequently conducted an unbiased proteomic screen using antibody-based affinity purification (*20*) in conjunction with mass spectrometry. Through our proteomic analysis, we identified proteins that directly or indirectly associate with the functional GFP-tagged PILS2, PILS3, and PILS6 fusion proteins. Notably, components of the ERAD complex, including HMG-CoA Reductase Degradation 1A (HRD1A), HRD1B, and the Proteins Associated with HRD1-1 (PAWH1 and PAWH2), emerged as the most abundant interactors of PILS2, PILS3, and PILS6 (Fig. 2A and table S1). In addition, although other ERAD components like DERLIN1 (DER1), DER2.1, and DER2.2 were detected in most biological replicates, their presence was not consistent across all samples (table S1). This set of data suggests that PILS proteins can associate with key components of the ERAD complex.

To further validate our proteomic findings, we coexpressed PILS3-GFP alongside ERAD components, such as PAWH1/2 and DERs, tagged with mScarlet for coimmunoprecipitation (co-IP) in *Nicotiana benthamiana*. Our results demonstrated a strong association between PILS3-GFP and both PAWH1-mScarlet and PAWH2-mScarlet, as well as with DER2.1-mScarlet and DER2.2-mScarlet (Fig. 2B). Using an HRD1 antibody that recognizes endogenous HRD1A/B in *Arabidopsis thaliana*, we further confirmed the interaction of PILS6-GFP with HRD1 in stable transgenic plants (Fig. 2C and fig. S2A). Collectively, these findings support the notion that PILS proteins interact with the ERAD complex.

To investigate whether PILS proteins directly interact with ERAD components, we used a heterologous system, using a mating-based split-ubiquitin system for transmembrane proteins in yeast (*21*). This analysis suggested direct interactions between PILS3 and HRD1A/B, as well as with DERLIN proteins like DER1 and DER2.1 (Fig. 2D). However, PILS3 did not interact with the regulatory ERAD component EBS5/HRD3, indicating specificity in the observed interactions (Fig. 2D). To consolidate these possibly direct interactions in plant cells, we used ratiometric bimolecular fluorescence complementation (rBiFC) (*22*), which allows the normalization of interaction-dependent signals against a second fluorescent reporter expressed from the same transferred DNA (T-DNA). Using this system, we obtained significant interaction signals between PILS3-cYFP and HRD1B-nYFP, DER2.1-nYFP, as well as DER2.2-nYFP when coexpressed in *N. benthamiana* (Fig. 2E and fig. S2, B and C).

To further consolidate this interaction under nonstressed conditions in planta, we performed a co-IP using anti-HRD1 antibodies on wild-type *Arabidopsis* lysates, followed by label-free quantitative mass spectrometry. Mock IPs (beads without antibody) were used to assess background binding. This approach confirmed strong enrichment of several canonical ERAD components when compared to control, including HRD1A/B itself as well as EBS5, EBS7, PAWH1, and PAWH2 (fig. S2D and table S3). Gene set enrichment analysis of the quantified proteins showed an enrichment of proteins related to ERAD and proteolysis. Notably, native PILS5, as well as DER2.1 and DER2.2, were found among the significantly enriched proteins (fig. S2D).

This suggests that PILS proteins can directly interact with ERAD complex components under physiological, nonstressed conditions, categorizing them as potential physiological clients of the ERAD system.

The ERAD pathway can target functional proteins, but its canonical role is to identify, extract, and degrade misfolded or aberrant proteins from the ER. We were initially concerned that PILS fusion proteins may be recognized by this quality control mechanism, which may engage with the ERAD pathway. This led us to address whether the PILS/ERAD interactions impose quantitative constraints on the efficient processing of misfolded substrates within this pathway. To explore such a scenario, we used the *bri1-5* mutant, which encodes a structurally imperfect yet functional brassinosteroid receptor. In this mutant, BRI1-5 proteins undergo constitutive degradation via the ERAD pathway (*23*). Both pharmacological and genetic interference with ERAD function can reduce the degradation of the BRI1-5 receptor, thereby promoting its secretion to the plasma membrane and partially rescuing the dwarfed phenotype characteristic of this mutant (*23*, *24*).

To quantitatively assess BRI1-5 processing by the ERAD pathway, we performed the well-established Endoglycosidase H (Endo H) assay, which distinguishes the size differences between plasma membrane–localized BRI1 and its ER-localized counterpart based on differences in glycan modifications during transit through the secretory pathway (*23*, *24*). Notably, neither PILS6 overexpression nor loss-of-function mutations in *pils6* affected BRI1-5 processing (fig. S3A), suggesting that BRI1-5 remains retained in the ER. Furthermore, the root phenotype of the *bri1-5* mutant, along with the constitutive degradation of BRI1-5, was not significantly altered in these lines (fig. S3, B to D). These findings indicate that overexpression of PILS6-GFP does not interfere with the ERAD-dependent degradation of BRI1-5. Consequently, we conclude that the interaction of PILS6-GFP with ERAD components is unlikely to negatively affect their canonical function.

**Fig. 3.**
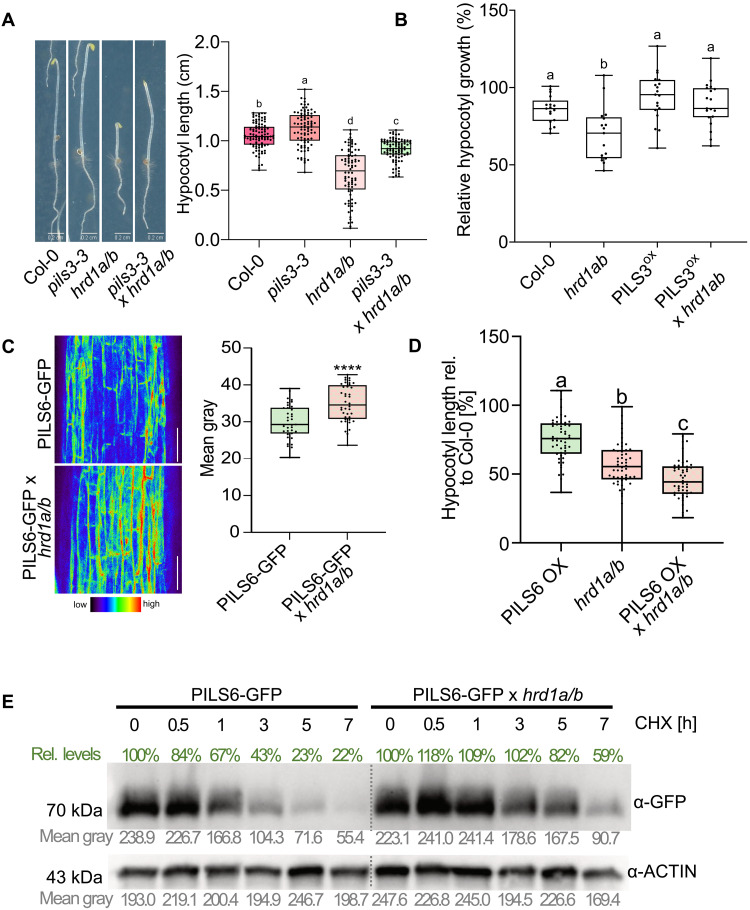
HRD1 activity defines PILS turnover and hypocotyl growth. (**A**) Dark-grown hypocotyl length was measured for Col-0 wild type, *pils3-3*, *hrd1a/b*, and *pils3-3 hrd1a/b* mutants. *n* = 50, one-way ANOVA followed by Tukey’s multiple comparison test (letters indicate statistical differences: *P* < 0.001). (**B**) Relative dark-grown hypocotyl growth (normalized to respective MOCK treatment) of Col-0 wild type, *hrd1a/b*, *p35S::PILS3* overexpressors (PILS3 OX), and PILS3 OX in *hrd1a/b* mutants. Seedlings were germinated on ½ MS plates for 3 days and then transferred to synthetic auxin (NAA, 750 nM) or solvent (DMSO) containing plates for 2 days. *n* = 20, one-way ANOVA followed by Tukey’s multiple comparison test (letters indicate statistical differences: *P* < 0.001). (**C**) Representative images and quantifications of PILS6-GFP and PILS6-GFP x *hrd1a hrd1b* signal in 3-day-old dark-grown seedlings. Scale bars, 50 μm. *n* = 35 to 40, Student’s *t* test (****P < 0.0001). (**D**) Relative hypocotyl length of 5-day-old dark-grown seedlings. *n* = 45 to 60, one-way ANOVA followed by Tukey’s multiple comparison test (b, c: *P* < 0.0001). (**E**) Immunoblot analysis of the PILS6-GFP in wild-type or *hrd1a hrd1b* background. Four-day-old seedlings were treated for the indicated time with 100 μM CHX in liquid ½ MS, and proteins were separated by SDS-PAGE and analyzed by immunoblotting using α-GFP antibodies. α-Actin antibody was used for normalization. Values below each band indicate the mean gray value, and the percentage changes relative to the loading control are shown above each band. All panels with box plots: Box limits represent the 25th percentile and 75th percentile; horizontal line represents the median. Whiskers display min. to max. values. All experiments were repeated at least three times.

Although the HRD1 branch of the ERAD pathway is well recognized for its role in maintaining ER quality control in plants, it also targets physiological substrates in yeast and animal cells. These physiological ERAD clients are functional proteins that are conditionally degraded to fine-tune their activities (*25*). However, the existence of physiological clients of ERAD complexes in plants are just surfacing (*26*–*28*), hence remaining an exciting area of research. Moreover, physiological HRD1 clients remain largely unknown in plants (*29*). We found that the *hrd1a hrd1b* double mutants exhibited reduced hypocotyl growth under dark conditions, suggesting that the ERAD complex could play a physiological role in regulating growth beyond its traditional function in ER quality control (Fig. 3A). The PILS proteins redundantly limit organ growth, with the *pils3* single mutants already demonstrating accelerated hypocotyl growth compared to wild-type plants (*2*). The *pils3 hrd1a hrd1b* triple mutant partially alleviated the growth defects observed in the *hrd1a hrd1b* double mutants (Fig. 3A). Moreover, auxin-responsive genes were down-regulated in the *hrd1a hrd1b* mutant (fig. S3E) and its hypocotyl growth was hypersensitive to the exogenous application of auxin when compared to wild type (Fig. 3B). This set of data suggests deregulated auxin-dependent responses and growth in the *hrd1a hrd1b* double mutant. Notably, the hypersensitivity to exogenous auxin could be compensated by *PILS3* overexpression. This set of data reveals that HRD1 contributes to plant growth regulation under nonstressed conditions. Although additional growth-related defects from *hrd1a hrd1b* mutations cannot be ruled out, our findings suggest that PILS proteins contribute to HRD1-reliant growth control.

Our previous work on PILS6 proteins revealed a conditional posttranslational control of PILS proteins (*3*). To investigate whether the ERAD complex influences the posttranslational regulation of PILS proteins, we hence introduced the constitutive PILS6-GFP expressing lines with the *hrd1a hrd1b* double mutants. The genetic interference with *HRD1* function led to a quantitative increase in PILS6-GFP abundance in dark-grown hypocotyls (Fig. 3C). This suggests that HRD1 is crucial for determining the levels of PILS proteins under nonstressed conditions. Furthermore, the enhanced abundance of PILS6 in the *hrd1a hrd1b* mutant resulted in stronger growth inhibition of dark-grown hypocotyls compared to parental lines (Fig. 3D). These observations suggest that interference with the ERAD pathway stabilizes functional PILS proteins, thereby enhancing growth repression.

Next, we used Western blotting as a semiquantitative method, using a cycloheximide (CHX) chase experiment to assess protein turnover. CHX inhibits de novo protein synthesis, allowing us to monitor the degradation dynamics of PILS6-GFP. Following CHX treatment, PILS6-GFP levels steadily declined over time (Fig. 3E). Notably, this decline was markedly reduced in the *hrd1a hrd1b* double mutant background, indicating that HRD1 activity contributes to the turnover of PILS6-GFP.

To further examine whether HRD1 has a direct impact on PILS turnover, we aimed to pharmacologically inhibit HRD1 using LS102, which selectively disrupts HRD1 activity in animal cells (*30*). As mentioned above, the *hrd1a hrd1b* mutants displayed reduced hypocotyl growth in darkness (Fig. 3, A, B, and D), and this phenotype was phenocopied by LS102 application on wild-type Col-0 plants (fig. S3F). Notably, the LS102-induced effects on hypocotyl expansion were abolished in the *hrd1a hrd1b* double mutants (fig. S3F), indicating that LS102 specifically targets HRD1-dependent processes in dark-grown hypocotyls of *Arabidopsis*.

**Fig. 4.**
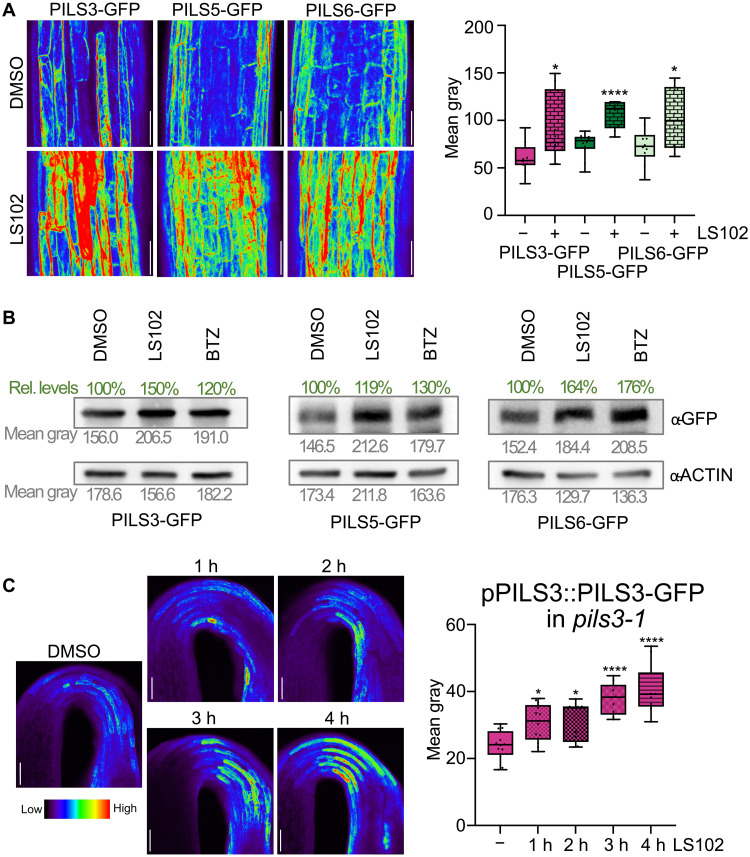
Inhibition of HRD1 has an immediate effect on PILS protein stability. (**A**) Representative images and quantifications of GFP-PILS3, PILS5-GFP, and PILS6-GFP signal in 3-day-old dark-grown seedlings. Seedlings were grown on solid ½ MS and treated with DMSO or 5 μM LS102 (HRD1 inhibitor) in liquid ½ MS for 4 hours. Scale bars, 50 μm. *n* = 10 to 12, Student’s *t* test between DMSO and LS102. (**B**) Immunoblot of 4-day-old dark-grown GFP-PILS3, PILS5-GFP, and PILS6-GFP seedlings treated with DMSO, 10 μM LS102, or 50 μM BTZ for 4 hours in liquid ½ MS. Anti-Actin antibody was used for normalization. Values below each band indicate the mean gray value (in gray), and the percentage changes relative to the loading control are shown above each band (in green). (**C**) Representative images and quantifications of pPILS3::PILS3-GFP in the *pils3-1* background in 3-day-old dark-grown seedlings. Seedlings were grown on solid ½ MS and treated with DMSO or 5 μM LS102 in liquid ½ MS for 1 to 4 hours. *n* = 10, one-way ANOVA followed by Tukey’s multiple comparison between DMSO and LS102. In all panels with box plots: Box limits represent the 25th percentile and 75th percentile; horizontal line represents the median. Whiskers display min. to max. values. *P* values: *P < 0.05 and ****P < 0.0001. All experiments were repeated at least three times. h, hours.

Consistent with the genetic interference, pharmacological inhibition of HRD1 significantly increased the protein levels of GFP-PILS3, PILS5-GFP, and PILS6-GFP, which is reminiscent of the inhibition of the proteasome (Fig. 4, A and B). Moreover, blocking HRD1 function also resulted within hours in a relatively fast increase in the abundance of functional pPILS3::PILS3-GFP (Fig. 4C). In contrast, *PILS3* expression was not affected during this experimental condition (fig. S1E). Collectively, these results suggest that the HRD1-reliant ERAD complex has a direct role in modulating the physiologically relevant levels of PILS proteins.

To genetically increase ERAD activity, we turned to EBS5/HRD3 because it plays a conserved role in enhancing HRD1 activity (*24*, *31*). Notably, the introduction of an additional copy of pEBS5:EBS5 leads to the mild up-regulation of *EBS5* transcript (fig. S3G). Up-regulation of EBS5 correlated with reduced PILS protein levels, such as in the constitutive p35S::PILS5-GFP expression line (fig. S4A). This reduction in PILS5 levels correspondingly balanced the dark-grown hypocotyl phenotype of PILS5 overexpressing seedlings (fig. S4B), suggesting that the degraded PILS5 proteins were functional.

Our findings collectively reveal that the ERAD pathway directly regulates the turnover of functional PILS proteins under nonstressed conditions, ultimately influencing organ growth. To further validate this role, we next explored the requirement of HRD1 for the conditional control of PILS turnover. We previously demonstrated that both internal and external signals, such as the phytohormone brassinosteroid and elevated ambient temperatures, induce the turnover of at least PILS1, PILS2, PILS3, PILS5, and PILS6 proteins in roots, affecting auxin-dependent growth (*3*, *5*).

To investigate the conditional turnover of PILS proteins in the main roots, we applied brassinolide (BL) (fig. S4C) or transferred constitutively PILS6-GFP expressing plants to elevated temperatures (fig. S4D). As expected, both BL treatment and high temperature induced the PILS6 turnover in the wild type. The conditional induction of PILS6 degradation was, however, significantly reduced in *hrd1a hrd1b* mutants (fig. S4, C and D), which also affected the respective root organ growth rates (*3*, *5*) (fig. S4, E and F). This suggests that the ERAD complex regulates the conditional turnover of functional PILS proteins.

We previously revealed that ER-stress responses can modulate PILS proteins in a posttranslational manner (*6*). Notably, both brassinosteroid application and high temperatures intersect with ER stress responses (*32*, *33*) and hence may have a general impact on ER homeostasis. Thus, we next aimed to identify specific conditions under which individual PILS proteins are selectively regulated without globally triggering the ER stress pathway. Given that changes in PILS protein turnover may alleviate root growth repression (*3*, *5*), we used root growth dynamics in overexpression lines of PILS2, PILS3, PILS5, and PILS6 to screen for selective modulators of PILS protein turnover.

**Fig. 5.**
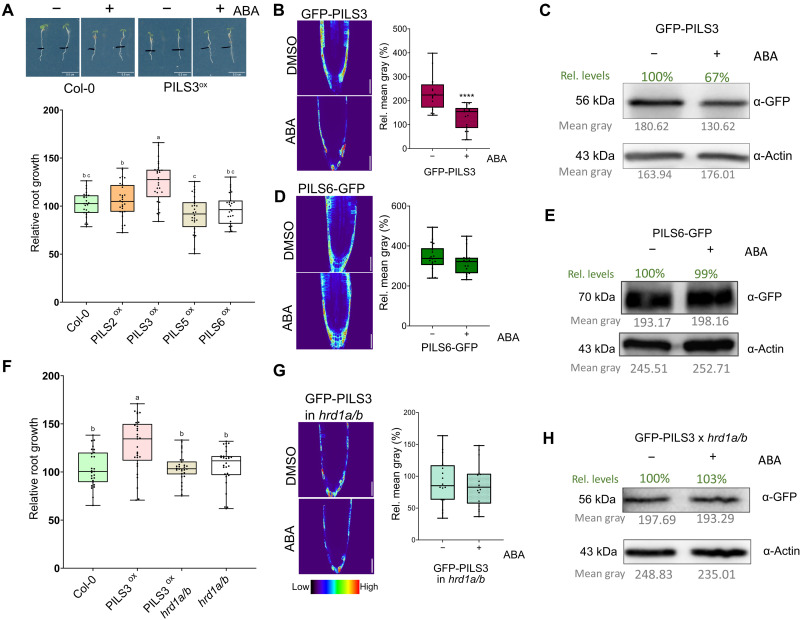
HRD1 regulates conditional PILS3 turnover. (**A**) Representative images and relative root growth quantification of 3-day-old seedlings, which were transferred for 1 day on ½ MS media containing DMSO or 100 nM ABA. *n* = 28 to 31, one-way ANOVA followed by Tukey’s multiple comparison test (letters indicate statistical differences: *P* < 0.0001). (**B**, **C**, **D**, **E**, **G**, and **H**) Representative images, its quantifications [(B), (D), and (G)], and immunoblots [(C), (E), and (H)] of GFP-PILS3 [(B) and (C)], PILS6-GFP [(D) and (E)], and GFP-PILS3 in hrd1a/b [(G) and (H)] signal in 4-day-old seedlings. Seedlings were grown on solid ½ MS and treated with DMSO or 100 nM ABA in liquid ½ MS for 4 hours. Scale bars, 50 μm. *n* = 16 to 20 from replicates pooled after confirming consistency across experiments, Student’s *t* test between DMSO and ABA. Student’s *t* test (****P < 0.0001). Proteins were separated by SDS-PAGE and analyzed by immunoblotting using α-GFP, and α-Actin antibody was used for normalization; values below each band (in gray) indicate the mean gray value, and the percentage changes relative to the loading control are shown above each band (in green). Seedlings were grown for 4 days on solid ½ MS and treated with DMSO or 100 nM ABA for 4 hours. (**F**) Relative root growth quantification of 3-day-old seedlings, which were transferred for 1 day on ½ MS media containing DMSO or 100 nM ABA. *n* = 28 to 31, one-way ANOVA followed by Tukey’s multiple comparison test (a: *P* < 0.001). In all panels with box plots: Box limits represent the 25th percentile and 75th percentile; the horizontal line represents the median. Whiskers display min. to max. values. All experiments were repeated at least three times.

Considering the previously reported influences of auxin and brassinosteroids on PILS abundance (*5*, *7*), we first tested other phytohormones for a potential impact on PILS protein stability. We found that the application of low concentrations of the phytohormone abscisic acid (ABA) specifically rescued the short root phenotype of PILS3-overexpressing lines, whereas PILS2, PILS5, and PILS6 overexpressors and wild-type roots were largely not affected in these low hormone concentrations (Fig. 5A). In agreement with this observation, these low doses of ABA led to a reduction in the protein abundance of PILS3, whereas PILS6-GFP showed no detectable changes (Fig. 5, B to E). In agreement with a specific effect on PILS3, ER-stress-induced genes remained inactive under these experimental conditions (fig. S5, A and B). This set of data reveals that low doses of ABA specifically induce the turnover of PILS3 in an ER-stress-independent manner.

Next, we tested whether the proteasome and HRD1 are required for the ABA-induced degradation of PILS3. Using initially a pharmacological approach, we revealed that the ABA-induced down-regulation of PILS3 was blocked by the inhibition of the proteasome and HRD1 (fig. S6, A and B), suggesting an ERAD-dependent regulation. In agreement, the ABA-dependent regulation of PILS3, along with the differential root growth responses in PILS3 overexpressors, was abolished in the *hrd1a hrd1b* mutant background (Fig. 5, F to H). Accordingly, we conclude that ABA specifically induces PILS3 degradation in an ERAD-dependent manner. These findings highlight the necessity of the HRD1-reliant ERAD for the selective degradation of PILS proteins.

Collectively, our data indicate that functional PILS proteins are conditionally recruited to the ERAD complex even in the absence of ER stress conditions, initiating their proteasome-dependent turnover. Beyond its established role in ER quality control for plants, we propose that the HRD1-reliant ERAD complex also fulfills a crucial physiological role—regulating the conditional turnover of functional proteins and influencing plant growth regulation.

## DISCUSSION

In our previous work, we demonstrated that ER stress increases and decreases the stability of PILS proteins in a tissue-specific manner (*6*), contributing to auxin-dependent stress adaptation responses. However, the molecular machinery responsible for controlling PILS turnover remained undefined. In the present study, we now identify specific ERAD components—particularly the HRD1 E3 ligases—as interactors of PILS proteins as well as regulators of PILS turnover under nonstressed conditions. Endogenous PILS proteins are expressed at low levels, and here we mainly used functional PILS-GFP fusion constructs to monitor PILS protein dynamics. These reporter lines were previously validated to rescue the respective mutant phenotypes (*1*–*3*), providing suitable tools for analyzing PILS turnover and function in planta. We fully recognize the importance of distinguishing artifactual degradation of tagged proteins from bona fide ERAD-mediated turnover of physiological substrates. To ensure the relevance of our findings, we took multiple complementary measures: We initially used constitutive PILS-GFP expression lines to specifically address the posttranslational protein control in the *hrd1a hrd1b* mutant background. We additionally used pharmacological inhibition of proteasome (BTZ) and HRD1 (LS102) activity in functional pPILS3::PILS3-GFP lines, thus confirming these effects under endogenous expression levels. Moreover, we specifically tested whether PILS overexpression leads to unspecific ERAD activation by using the *bri1-5* misfolded receptor allele as a reporter. We observed no evidence for ER stress induction or global ERAD interference, arguing against saturation or non-specific engagement of the ERAD machinery. Our conclusions are further supported by converging independent data where genetic (*hrd1a hrd1b*), pharmacological (LS102), and physiological (ABA, BR, and heat) cues all consistently alter PILS turnover in a proteasome-dependent manner and modulate auxin-responsive growth outcomes. Moreover, *hrd1a hrd1b* double mutants are defective in auxin responses and growth, which is genetically modulated by changes in *PILS* activity. The selective destabilization of PILS3 under ABA treatment, whereas other PILS members remain unaffected, reveals a context-specific ERAD targeting mechanism, not generally attributable to artifacts of GFP fusion proteins. Together, our findings provide accumulating functional, genetic, and biochemical evidence that PILS proteins are physiological clients of the HRD1-dependent ERAD pathway, extending its function in plants beyond canonical quality control to include developmentally relevant hormone transport regulation.

The identification of PILS proteins as physiological clients of the ERAD complex opens previously undefined avenues for understanding how plants regulate ER protein levels in response to various internal and external stimuli. Notably, HRD1-dependent ERAD pathways have been suggested to selectively target properly folded proteins for degradation in nonplant systems, thereby fine-tuning their physiological levels (*25*). Our findings suggest that HRD1 plays an additional role in plant growth coordination. We propose that this additional role of ERAD has profound implications for understanding how plants adapt to fluctuating physiological conditions. Given that PILS proteins are involved in gating nuclear signaling of auxin—a key hormone influencing growth and development—the regulation of their turnover directly affects auxin availability and homeostasis (*1*–*3*). Thus, the ability of the ERAD complex to modulate PILS levels could provide a general mechanism through which plants respond to internal and external cues. Although the possibly widespread developmental contributions need to be further explored, our findings highlight that ERAD-mediated turnover of PILS proteins is conditional and can be modulated by internal and environmental signals. Such a dynamic adjustment process can optimize plant growth and development, which becomes increasingly important in the face of environmental challenges. It needs to be addressed to what degree other E3 ligases within the ERAD pathway may recognize PILS as physiological targets. This could potentially provide functional redundancy in ER homeostasis and further enhance plant resilience.

The conditional degradation of physiological clients, such as PILS proteins, also raises intriguing questions about substrate recognition by the ERAD complex. Although the canonical function of HRD1 is to identify, extract, and degrade misfolded or aberrant proteins from the ER (*34*, *35*), the identification of physiologically relevant clients like PILS suggests that the pathway discerns substrates based on regulatory cues. The specific mechanisms through which PILS proteins are recognized by the ERAD machinery remain to be uncovered but could provide fundamental insights into the activity regulation and/or substrate recognition of ERAD components. We propose that the specific, ABA-dependent regulation of PILS3 could function as a suitable model to study the selectivity of HRD1-reliant ERAD activation, which warrants further investigations.

In conclusion, our work transforms our view on HRD1 and ERAD function in plants, affecting the integration of hormone signaling, stress responses, and ER homeostasis, unlocking previously unknown perspectives on plant growth regulation.

## MATERIALS AND METHODS

### Plant material and growth conditions

The following plant materials were used: *A. thaliana* Col-0 (wild type), p35S::GFP-PILS2 (*1*), p35S::GFP-PILS3 (*2*), p35S::PILS5-GFP (*1*), p35S::PILS6-GFP (*3*), pPILS3::PILS3-GFP in *pils3-1* (*2*), *pils6-1* (*3*), p35S::DER1-mScarlet (*6*), and *bri1-5* (*36*). Seeds were stratified at 4°C for 2 days in the dark. Seedlings were grown vertically on half-strength Murashige and Skoog medium [½ MS salts (Duchefa), pH 5.9, 1% sucrose, and 0.8% agar]. Plants were grown under long-day (16-hours light/8-hours dark) or under dark conditions at 20° to 22°C.

### Genotyping the *hrd1a hrd1b* double mutant

*hrda1* and *hrd1b* single mutants were obtained from the Nottingham Arabidopsis Stock Centre (SALK_032914 and SALK_061776), crossed, and genotyped using primers listed in table S2.

### Chemicals and treatments

BTZ (Santa Cruz), LS102 (Merck), BFA (Merck), VAC1 (*15*), FM4-64 (Merck), Endo H (Merck), CHX (Merck), ABA (Sigma-Merck), and tunicamycin (TM) (Santa Cruz) were all dissolved in dimethyl sulfoxide (DMSO) (Duchefa). Treatments with BTZ and LS102 were performed on 3- to 4-day-old seedlings (transferred to supplemented media) or germinated directly on the respective compound. Treatments with ABA and TM were performed on 3- to 4-day-old seedlings (treated or transferred to supplemented media).

### DNA constructs

The coding DNA sequence (CDS) of PILS3, DER2.1, DER2.2, PAWH1, and PAWH2 and the genomic sequence of EBS5 were amplified by polymerase chain reaction (PCR) (primers are listed in table S2) from cDNA using Q5 High-Fidelity DNA Polymerase (NEB) and cloned either under the 35*S* promoter together with mScarlet-I for DER2.1, DER2.2, PAWH1, and PAWH2, with GFP for PILS3 or for EBS5 under its own promoter into pPLV03 using Gibson Assembly (NEB).

For split ubiquitin and BiFC assay, the CDS of PILS3, HRD1A, HRD1B, DER1, DER2.1, DER2.2, and EBS5 were amplified by PCR (primers are listed in table S2) from cDNA using Q5 High-Fidelity DNA Polymerase (NEB) and cloned for split ubiquitin assay into pDONR221 and for rBiFC into pDONR221-L1L4/L3L2. Subsequently, the bait (PILS3) and preys were recombined into pMetYC-DEST (*37*) and PNX35-DEST (*38*), respectively, or into the rBiFC vector (*39*).

The resulting constructs were transformed into Col-0 plants using the floral dipping method (*38*) or for transient transformation in tobacco plants.

### Microscopy

Confocal microscopy was done with a Leica SP8 (Leica). Fluorescence signals for GFP (excitation, 488 nm; emission peak, 509 nm), mScarlet-i (excitation, 561 nm; emission peak, 607 nm), and yellow fluorescent protein (YFP) (excitation, 513 nm; emission peak, 527 nm) were detected with a 10x, 20x, or 40x (dry and water immersion, respectively) objective. *Z*-stacks were recorded with a step size of 840 nm. On average, 24 slices were captured, resulting in an average thickness of ~20 μm. Image processing was performed using LAS AF lite software (Leica) or ImageJ.

### Transient transformation

Transient transformation was performed as described by Castilho (*40*). Subcellular localization/protein extraction was performed 3 days after transformation.

### Protein extraction, IP, and immunoblot analysis

Seedlings or tobacco leaves were ground to fine powder in liquid nitrogen and solubilized with extraction buffer [25 mM Tris (pH 7.5), 10 mM MgCl_2_, 15 mM EGTA, 75 mM NaCl, 1 mM dithiothreitol (DTT), and 0.1% Tween 20, with freshly added proteinase inhibitor cocktail (Roche) and 40 mM l(+)-ascorbic acid for tobacco]. After spinning down for 60 min at 4°C with 20,000 rpm, the supernatant was transferred to a new tube and the protein concentration was assessed using the Bradford method. Protein extracts were either used directly for immunoblot with anti-GFP (Roche, #11814460001, 1:1,000), anti–red fluorescent protein (RFP) (ChromoTek, #6 g6, 1:1,000), anti-BRI (Agrisera, #AS12 1859, 1:1,000), anti-HRD1 (Invitrogen, #PA5-110899, 1:1,000), or anti-Actin (Sigma-Aldrich, #A0480, 1:10,000) and goat anti-mouse immunoglobulin G (IgG) (Jackson ImmunoResearch, #115-036-003, 1:10,000) for detection or for immunoprecipitation with anti-GFP beads (ChromoTek, #gtma) or anti-RFP beads (ChromoTek, #rtma) followed by either immunoblot or mass spectrometry. For the Endo H treatment, protein extracts were combined with 10x Glycoprotein Denaturing Buffer (NEB) and denatured by heating at 100°C for 10 min. The extracts were then combined with 10X GlycoBuffer 3 (NEB) and Endo H (NEB) and incubated at 37°C for 1 hour. The reaction was then analyzed via immunoblot.

### Mass spectrometry

Beads were transferred to new tubes and resuspended in 40 μl of 2 M urea in 50 mM ammonium bicarbonate (ABC). Disulfide bonds were reduced with 10 mM DTT for 30 min at room temperature before adding 25 mM iodoacetamide and incubating for another 30 min at room temperature in the dark. The remaining iodoacetamide was quenched by adding 5 mM DTT, and the proteins were digested with 150 ng of trypsin (Trypsin Gold, Promega) at room temperature for 2 hours. The supernatant was transferred to a new tube, the beads were washed with another 30 μl of 2 M urea in 50 mM ABC, and the wash was combined with the supernatant. After diluting to 1 M urea with 50 mM ABC, another 150 ng of trypsin was added and let digest overnight at 37°C in the dark. Digestion was stopped by adding trifluoroacetic acid (TFA) to a final concentration of 0.5%, and the peptides were desalted using C18 Stagetips (*41*).

Peptides were separated on an Ultimate 3000 RSLC nano-flow chromatography system (Thermo Fisher Scientific), using a pre-column for sample loading (Acclaim PepMap C18, 2 cm by 0.1 mm, 5 μm) and a C18 analytical column (Acclaim PepMap C18, 50 cm by 0.75 mm, 2 μm, both Thermo Fisher Scientific), applying a linear gradient from 2 to 35% solvent B (80% acetonitrile and 0.08% formic acid; solvent A 0.1% formic acid) at a flow rate of 230 nl/min over 120 and 60 min. Eluting peptides were analyzed on a Q Exactive HF-X Orbitrap mass spectrometer coupled to the LC via the Proxeon nanospray source, respectively, on an Orbitrap Exploris 480 with the FAIMS Pro interface coupled to the LC via the Nanospray Flex ion source (all Thermo Fisher Scientific). Coated emitter tips were obtained from MSWil (PepSep) and New Objectives.

### RNA extraction, cDNA synthesis, and quantitative PCR

RNA was extracted from 4-day-old seedlings if not stated otherwise using the InnuPREP Plant RNA Kit (Analytic Jena) according to the manufacturer’s instructions. Before cDNA synthesis, the RNA samples were treated with InnuPREP DNase I (Analytic Jena). One microgram of RNA was synthesized using FIREScript RT cDNA synthesis KIT (Solis BioDyne), and quantitative PCR was performed using 2x Takyon for SYBR Assay—no ROX (Eurogentec) following the manufacturer’s instructions on a CFX384 Touch Real-Time PCR Detection System (Bio-Rad). Primers used are listed in table S2, and the genes were normalized against UBQ5 and EIF4.

### Mass spectrometry data acquisition and analysis

The mass spectrometers were operated in data-dependent acquisition mode. For the Q Exactive HF-X measurements, survey scans were obtained in a mass range of 380 to 1650 mass/charge ratio (*m/z*) with lock mass activated, at a resolution of 120k at 200 *m/z* and an automatic gain control (AGC) target value of 3 × 10^6^. The 10 most intense ions were selected with an isolation width of 2.0 *m/z*, for max. 250 ms at a target value of 1 × 10^5^ and then fragmented in the higher-energy collisional dissociation (HDC) cell at 27% collision energy. The spectra were recorded in the Orbitrap at a resolution of 30k. Peptides with a charge of +1 or >+6 were excluded from fragmentation, the peptide match feature was set to preferred, the exclude isotope feature was enabled, and selected precursors were dynamically excluded from repeated sampling for 30 s.

Experiments on the Orbitrap Exploris were acquired with FAIMS using two compensation voltages (CVs) (−45 and −60 V) with 1.5-s cycle time. Full scans were obtained over the mass range of 350 to 1500 *m/z*, at a resolution of 60k and an AGC target of 100% with maximum injection time set to auto. Precursors of charge 2+ to 6+ were isolated in a 1 *m/z* window, with a maximum injection time of 100 ms for an AGC target of 100%, and subsequently fragmented applying a normalized collision energy of 28%. MS2 spectra were acquired in the Orbitrap at a 15k resolution. Selected precursors were dynamically excluded from repeated sampling for 20 seconds.

Raw data were processed using the MaxQuant software package (version 1.5.2.9 or 1.6.0.16) (*42*) and the TAIR10 protein database (December 2010, www.arabidopsis.org), the UniProt Arabidopsis reference proteome (release 2020-01, www.uniprot.org), as well as a database of the most common contaminants. The search was performed with full trypsin specificity and a maximum of two missed cleavages. Carbamidomethylation of cysteine residues were set as fixed, oxidation of methionine, phosphorylation of serine, threonine, and tyrosine and N-terminal acetylation as variable modifications. For label-free quantification, the “match between runs” feature and the label-free quantification (LFQ) function were activated—all other parameters were left at default.

Results were filtered at a false discovery rate of 1% at the protein and peptide spectrum match level. For downstream analysis, protein entries with only one razor and unique peptide and less than two quantification events were removed and missing LFQ values replaced by a constant.

### Split ubiquitin

For the split ubiquitin assay, yeast strains THY.AP4 and THY.AP5 were transformed with bait and prey constructs, respectively, using a modified protocol from (*43*). Approximately 100 μl of fresh yeast was scraped from YPD (yeast extract, peptone, and dextrose) plates and resuspended in 200 μl of sterile H_2_O. The resuspended yeast was then centrifuged for 5 min at 2000*g*, and the supernatant was removed. The yeast was then resuspended in 200 μl of yeast transformation buffer [40% PEG-3350 (polyethylene glycol, molecular weight 3350), 200 mM LiAc, and 100 mM DTT], 10 μl of single-stranded carrier DNA and 1 μg of plasmid DNA were added, and mixed by pipetting up and down. Yeast was incubated for 15 min at 30°C and for 45 min at 45°C. The cells were then plated on SD medium and incubated for 4 days at 28°C. A pool of transformed colonies was mated as described in (*37*). The selected diploid colonies were then incubated on plates containing selective medium (SD -Trp, -Leu, -Ade, -His, -Ura) at 21°C. Growth was recorded up to 9 days after plating.

### Statistical analysis and reproducibility

GraphPad Prism software 9 was used to evaluate the statistical significance of the differences observed between control and treated groups and to generate the graphs. All experiments were, if not stated different, always repeated at least three times, and the depicted data show the results from one representative experiment.

## Supplementary Materials

### The PDF file includes:

Supplementary Methods Figs. S1 to S6 Legends for tables S1 to S3 References
