# Supplementary material for: ERAD machinery controls the conditional turnover of PIN-LIKES in plants
Source: Sci Adv. 2025 Sep 19;11(38):eadx5027. doi: 10.1126/sciadv.adx5027 (PMC12448072; doi:10.1126/sciadv.adx5027)
Supplement: Supplementary file 1 — Supplementary Methods Figs. S1 to S6 Legends for tables S1 to S3 References [file sciadv.adx5027_sm.pdf]

Science Advances
AAAS

Supplementary Materials for

ERAD machinery controls the conditional turnover of PIN-LIKES in plants

Seinab Noura *et al.*

Corresponding author: Sascha Waidmann, sascha.waidmann@icloud.com;
Jürgen Kleine-Vehn, juergen.kleine-vehn@biologie.uni-freiburg.de

Sci. Adv. **11**, eadx5027 (2025)
DOI: 10.1126/sciadv.adx5027

The PDF file includes:

Supplementary Methods
Figs. S1 to S6
Legends for tables S1 to S3
References

Other Supplementary Material for this manuscript includes the following:

Tables S1 to S3

Supplementary Methods

Co-immunoprecipitation mass spectrometry analysis of Hrd1-interacting proteins

The Co-IP was performed essentially as described (*44*). The antibody against Hrd1 (Invitrogen, 17138823) was coupled to M-270 Epoxy Dynabeads at a ratio of 3 µg/mg beads using the Dynabeads®Antibody Coupling Kit (#14311D, life technologies) as recommended by the manufacturer. As control, non-coated beads were used. For each condition (pulldown: beads coated with anti-Hrd1; control: non-coated beads) 4 replicates were employed. Beads were prepared by washing in 500 µL IP-buffer. Plant material (300 mg) was ground in liquid nitrogen and dissolved in1.5 mL IP buffer containing 25 mM HEPES-KOH, pH 7.5, 2 mM EDTA, 100 mM NaCl, 0.5% Triton X-100 and 1% Plant Protease Inhibitor Cocktail (P9599, Sigma Aldrich). Samples were incubated in an Ultrasound bath for 10 min at 4-8°C and cell debris was removed by centrifugation at 20.000xg for 30 min at 4°C. The supernatant was transferred into fresh reaction tubes containing the prepared beads. Samples were incubated for 1h at 4-8°C under constant head-over rotation. The supernatant was removed and the beads were washed with 1 mL Wash Buffer I (25 mM HEPES-KOH, pH 7.5, 2 mM EDTA, 100 mM NaCl) and once with 500 µL afterwards. Beads were dissolved in 500 µL Wash Buffer II (10 mM Tris-HCl, pH 7.5, 150 mM NaCl, 0.5 mM EDTA), transferred to fresh tubes and washed again with 1 mL Wash Buffer II. The supernatant was discarded and the beads were dissolved in 35 µL elution buffer (50 mM Tris HCl, pH 7.5, 4% SDS, 10 mM DTT) and denatured at 70°C for 10 min. The supernatant was transferred to a fresh tube and the remaining beads were washed once again with 30 µl elution buffer. The supernatant was added to the prior and alkylation was performed by adding 3 µL chloroacetamide solution (1M stock) and incubation in the dark for 20 min at RT.

Sample purification and tryptic digestion were performed on a Hamilton STARlet automated liquid handling system (Hamilton) using a custom SP3 bead-based purification protocol (*45*). Samples were transferred to a 96-deep-well plate (Storage plate 96 well, 1 mL, Agilent Technologies) and magnetic bead separation was conducted using a 96-well magnet plate (Magnum Flex, Alpaqua). Proteins were bound to Sera-Mag SpeedBead magnetic carboxylate-modified particles (Cytiva) by incubating the samples with 100 µg of beads in 80% ethanol for 20 minutes at room temperature. After binding, samples were washed three times with 80% ethanol. Proteins were digested overnight at 37°C in 50 µL of 100 mM ammonium bicarbonate containing 0.1 µg of trypsin (Promega V5111, Madison, USA). Digestion was stopped the following day by adding 30 µL of 5% formic acid (FA). Peptides were desalted using self-packed SDB-RPS Stop and Go Extraction tips (*45*) (StageTips) composed of three layers of 1.0 × 1.0 mm (AttractSPE® Disks Bio RPS, AFFINISEP). The eluates were dried using a SpeedVac concentrator (Eppendorf) and reconstituted in 0.1% FA prior to loading onto Evotips (EV2013 Evotip Pure, Evosep), following the manufacturer's protocol.

For LC-MS analysis, an Evosep One system (Evosep) was coupled online to an Exploris 480 mass spectrometer (Thermo Fisher Scientific). Peptides were separated using a 44-minute

gradient (30 SPD workflow, Evosep) with a nano-LC column (EV1137, 15 cm × 150 µm performance column, evosep) maintained at 40°C using a column oven (PRSVO-V2, Sonation). Electrospray ionization was performed using a Nanospray Flex ion source (Thermo Fisher Scientific) with a stainless steel emitter featuring an integrated liquid junction (EV1072, Evosep) and mounted into an EasySpray adapter (EV1072, Evosep). A spray voltage of +2000 V was applied and the ion transfer tube was set to 275°C. Data were acquired in data-independent acquisition (DIA) mode. Each acquisition cycle consisted of one MS1 survey scan (RF lens: 40%, normalized AGC target: 300%, maximum injection time: 45 ms, m/z range: 350–1400, resolution: 120,000, profile mode) followed by MS2 fragment spectra (RF lens: 50%, normalized AGC target: 1000%, maximum injection time: 54 ms, resolution: 30,000, profile mode) acquired by higher-energy collision-induced dissociation (HCD) at a normalized collision energy of 28%. Fragment ions were collected across three m/z ranges: 6 windows of 14 *m/z* isolation width in a precursor *m/z* range from 361 to 450, 50 windows of 7 *m/z* isolation width in a precursor *m/z* range from 450 to 800 and 21 windows of 14 *m/z* isolation width in a precursor *m/z* range from 800 to 1100. Each isolation window overlapped by 1 m/z.

Raw data were parsed to mzML format using *MSConvert* (*46*) (V3.0.21229-9667f52) and database search was done using *FragPipe* (*47*) (V22.0) against an Araport11 database (*48*) concatenated by decoys and known contaminant sequences within Fragpipe. Precursor and fragment mass tolerance were set to ±20ppm with mass calibration and parameter optimization enabled. Variable modifications were oxidation of methionine (+15.9949 Da), N-terminal acetylation (+42.0106 Da), N-terminal cyclisation at glutamine or cysteine (-17.0265 Da) and glutamic acid (-18.0106 Da). Carbamidomethylation of cysteines was set as fixed modification (+57.02146 Da). Enzymatic specificity was set to trypsin (K|R) with no cuts after proline. Peptides between 7 and 35 amino acids in a mass range of 500 to 5000 Da were searched. Validation tools was enabled using *MSBooster* with *DIA-NN* models, *Percolator* (*49*) to validate PSMs and *PTMProphet* for validation of post-translational modifications. *ProteinProphet* (*50*) was enabled for protein inference. A spectral library generated from the *FragPipe* search was used for quantification with *DIA-NN* (*51*) within *FragPipe*. For analysis the protein groups file of the *DIA-NN* output containing LFQ (*47*) intensities as quantitative values was used. Data analysis, filtering and statistics were performed in *R*. Only proteins with non-zero LFQ values across all samples were used for statistical analysis. Log2 transformed LFQ intensities were used for linear modelling using the "robust" method of the *limma* (*52*) package in *R*. p-values obtained were corrected via multiple testing by the Benjamini and Hochberg approach (*53*). GSEA analysis was performed using annotations available from the *org.At.tair.db* package using *clusterProfiler* (*54*) in *R*.

Supplementary Figures

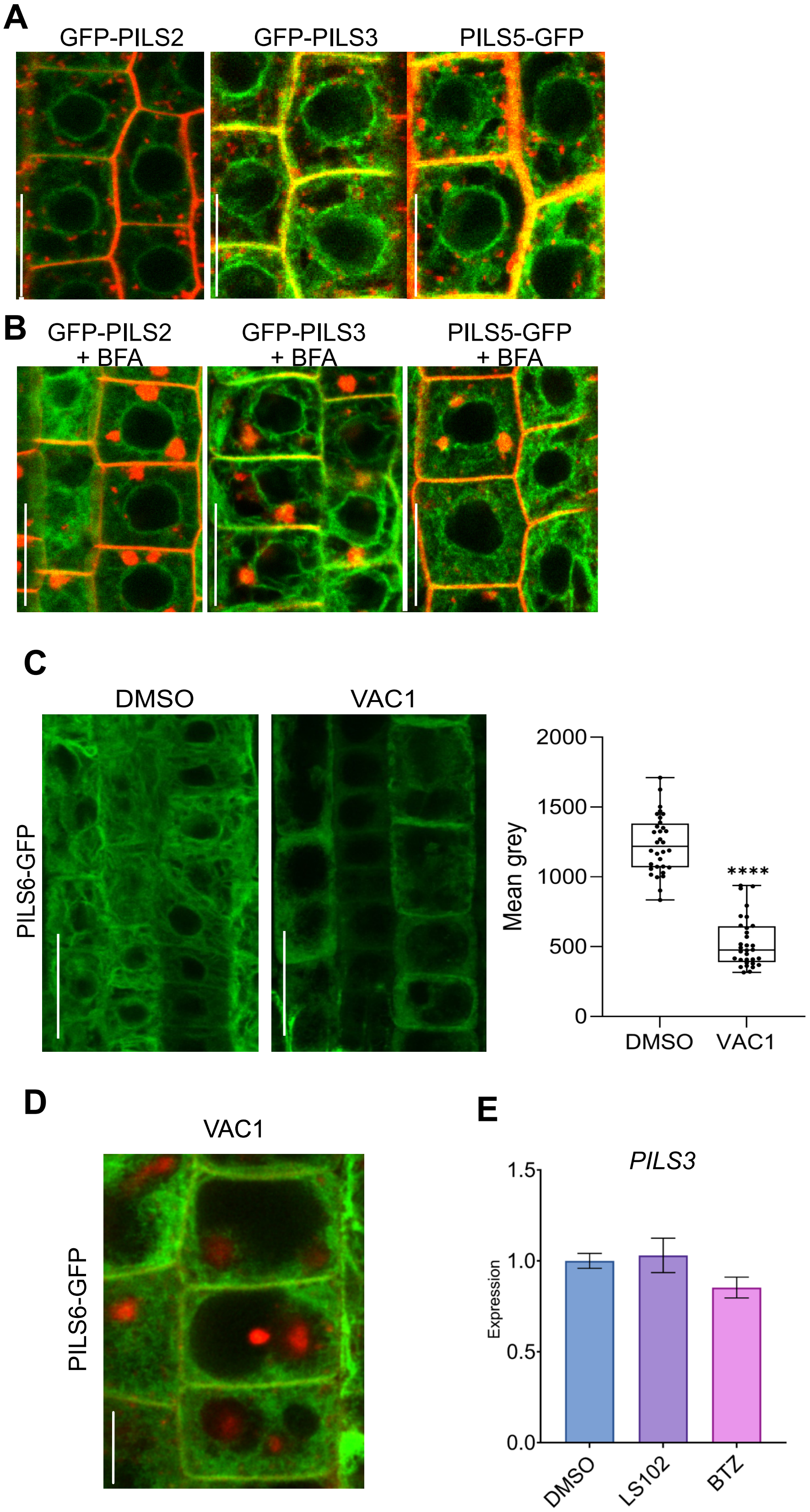

figure S1: PILS proteins are stably retained in the ER.

A-B, Representative images of untreated (A) and Brefeldin A (BFA)-treated (50 µM for 2h) (B) GFP-PILS2, GFP-PILS3 and PILS5-GFP. The endocytic dye FM4-64 was used as a counterstain and to illustrate BFA-induced endomembrane accumulations. Scale bars, 25 µm. **C,** Representative images and quantification of PILS6-GFP signal in roots, which were treated with solvent control (DMSO) or 10 µM Vacuolar Affecting Compound 1 (VAC1) for 1h. Box limits represent the 25th percentile and 75th percentile; the horizontal line represents the median. Whiskers display min. to max. values. n > 26, Student's t-test (****P < 0.0001). **D,** PILS6 was absent from VAC1-induced accumulations as indicated by FM4-64 staining. Scale bars, 10 µm. Experiments were done in liquid ½ MS medium using 5-day-old seedlings. All experiments were repeated at least three times. **E,** qPCR analysis of GFP transcript levels in pPILS3::PILS3-GFP expressed in *pils3-1* background. Transcript levels were normalised against *UBQ5* and *EIF4* in 4-day-old dark-grown hypocotyls after being treated with 5 µM LS102 or 40 µM BTZ for 1 hour. Bars represent means ± SD, n = 3.

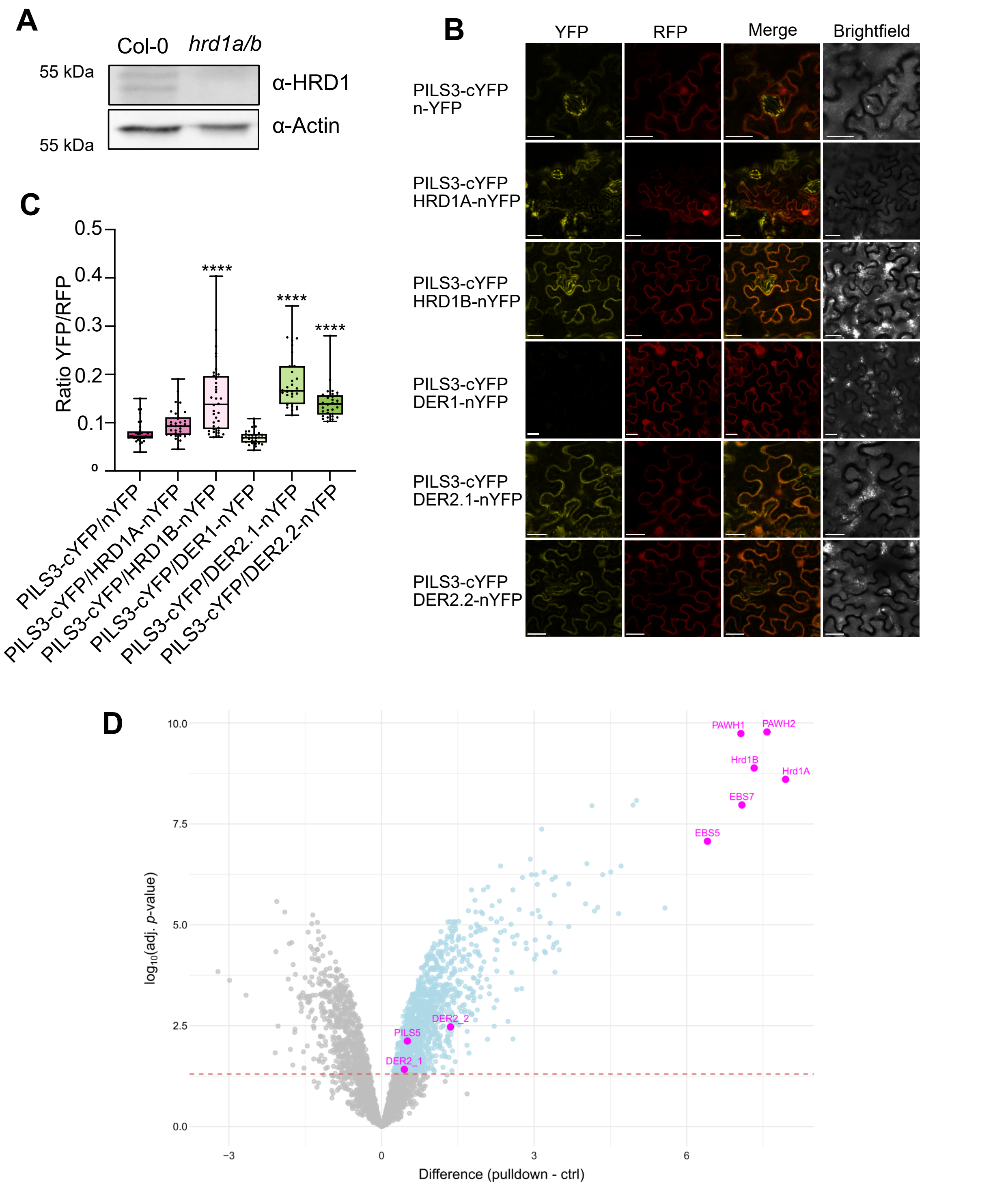

figure S2: PILS3 directly interacts with ERAD complex components.

A, Immunoblot of 7-days-old Col-0 and hrd1a hrd1b seedlings. Proteins were separated by SDS-PAGE and analysed by immunoblotting using -HRD1 antibodies. -Actin antibody was used for normalization. **B,** Ratiometric BioFluorescence Complementation (rBiFC) in Nicotiana benthamiana leaves transiently transformed with constructs encoding PILS3-cYFP and HRD1A-, HRD1B-, DER1-, DER2.1-, DER2.2-nYFP, or nYFP alone. A constitutively expressed mRFP (from the same T-DNA) was used as expression control (30). Scale bars, 25 µm. **C,** Quantification of BiFC signal was achieved by calculating the ratio between complemented YFP to RFP from 6 entire individual images and from 5 individual interfaces of two to three cells to avoid bias arising from picking single cells. One-way ANOVA followed by Tukey's multiple comparison test (****$P < 0.0001$). Box limits represent the 25th percentile and 75th percentile; the horizontal line represents the median. Whiskers display min. to max. values. All experiments were repeated at least three times. **D,** Volcano plot of IP-MS analysis showing the difference between log2 values of normalized label-free quantitation intensity values (LFQ) as a measure of protein abundance, plotted against the negative log10 of the adjusted limma-moderated p-value. Blue, proteins significantly co-enriched with Hrd1 (limma-moderated p-value < 0.05 after adjustment for multiple hypothesis testing), indicating potential interactors. Hrd1 and selected proteins of interest are depicted in magenta.

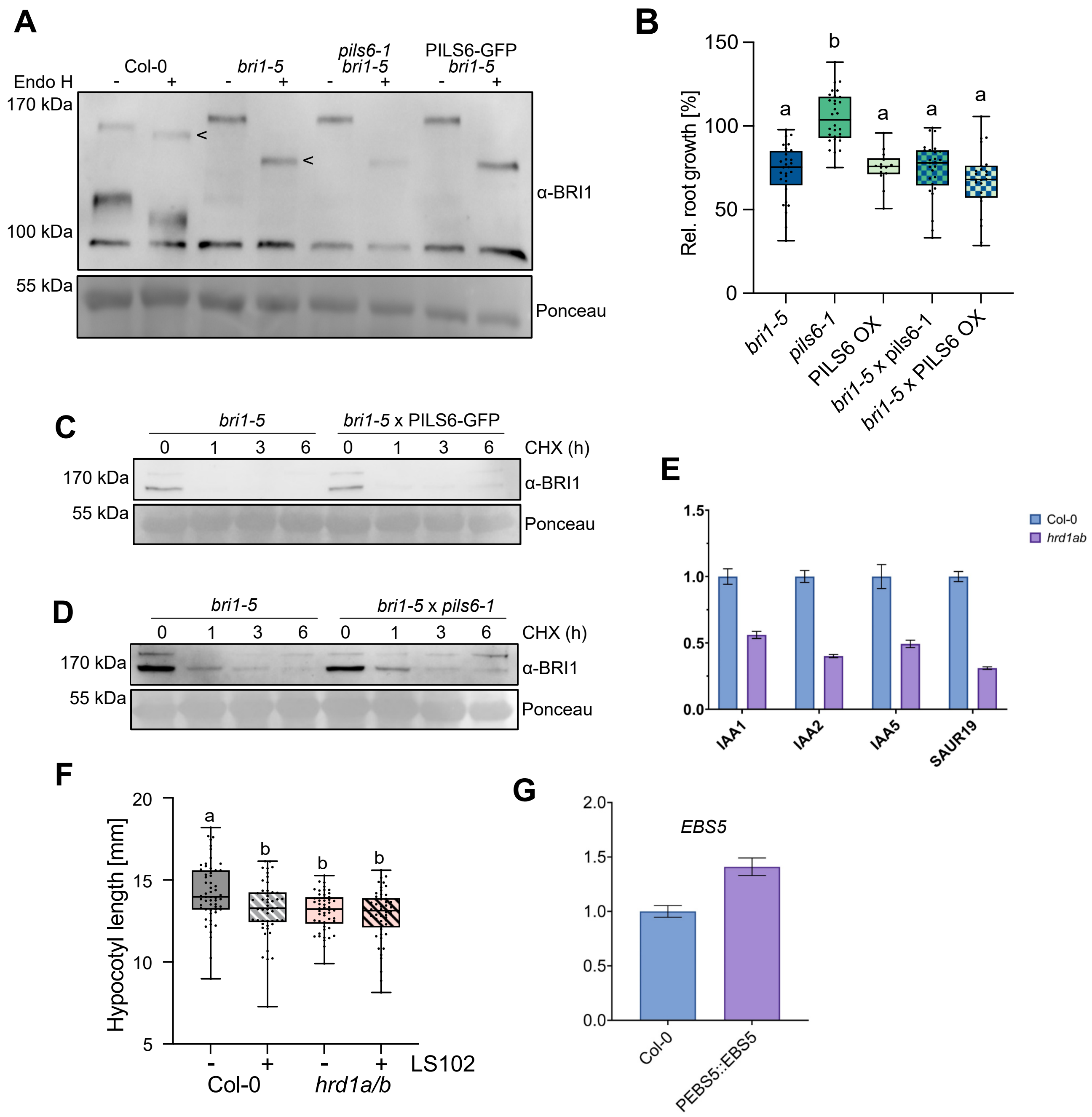

figure S3: PILS does not affect ERAD-dependent BRI1-5 processing.
A, Immunoblot of protein extracts from 7-day-old seedlings, treated with (+) or without (-) Endo H for 1 hour. Proteins were separated by SDS-PAGE and analysed by immunoblotting using -BRI1 antibodies. Ponceau staining was used for normalization. Arrowheads pinpoint size difference of PM and ER localised BRI1 receptors. **B,** Relative root length of 5-day-old seedlings. n = 15-30, One-way ANOVA followed by Tukey's multiple comparison test (b: $P < 0.001$). Box limits represent 25th percentile and 75th percentile; the horizontal line represents the median. Whiskers display min. to max. values. **C, D,** Immunoblot from 5-day-old seedlings treated for the indicated time with 100 µM cycloheximide (CHX) in liquid ½ MS. Proteins were separated by SDS-PAGE and analysed by immunoblotting using -BRI1 antibodies. Ponceau staining was used for normalization. **E,** qPCR analysis of auxin auxin-responsive genes detecting transcript levels of *IAA1*, *IAA2*, *IAA5*, and *SAUR19* in Col-0 and *hrd1a/b* mutants. Transcript levels were normalised against *UBQ5* and *EIF4*. 4-day-old dark-grown seedlings were used for RNA extraction. Bars represent means ± SD, n = 3. **F,** Hypocotyl length of 2-day-old dark-grown seedlings treated for 24h with DMSO or 10 µM LS102 in liquid ½ MS media. n = 50. one-way ANOVA followed by Tukey's multiple comparison test (b: $P < 0.001$). All experiments were repeated at least three times. **G,** qPCR analysis of *EBS5* transcript levels in Col-0 and pEBS5::EBS5 expressing transgenics. Transcript levels were normalised against *UBQ5* and *EIF4* in 2-week-old seedlings. Bars represent means ± SD, n = 3.

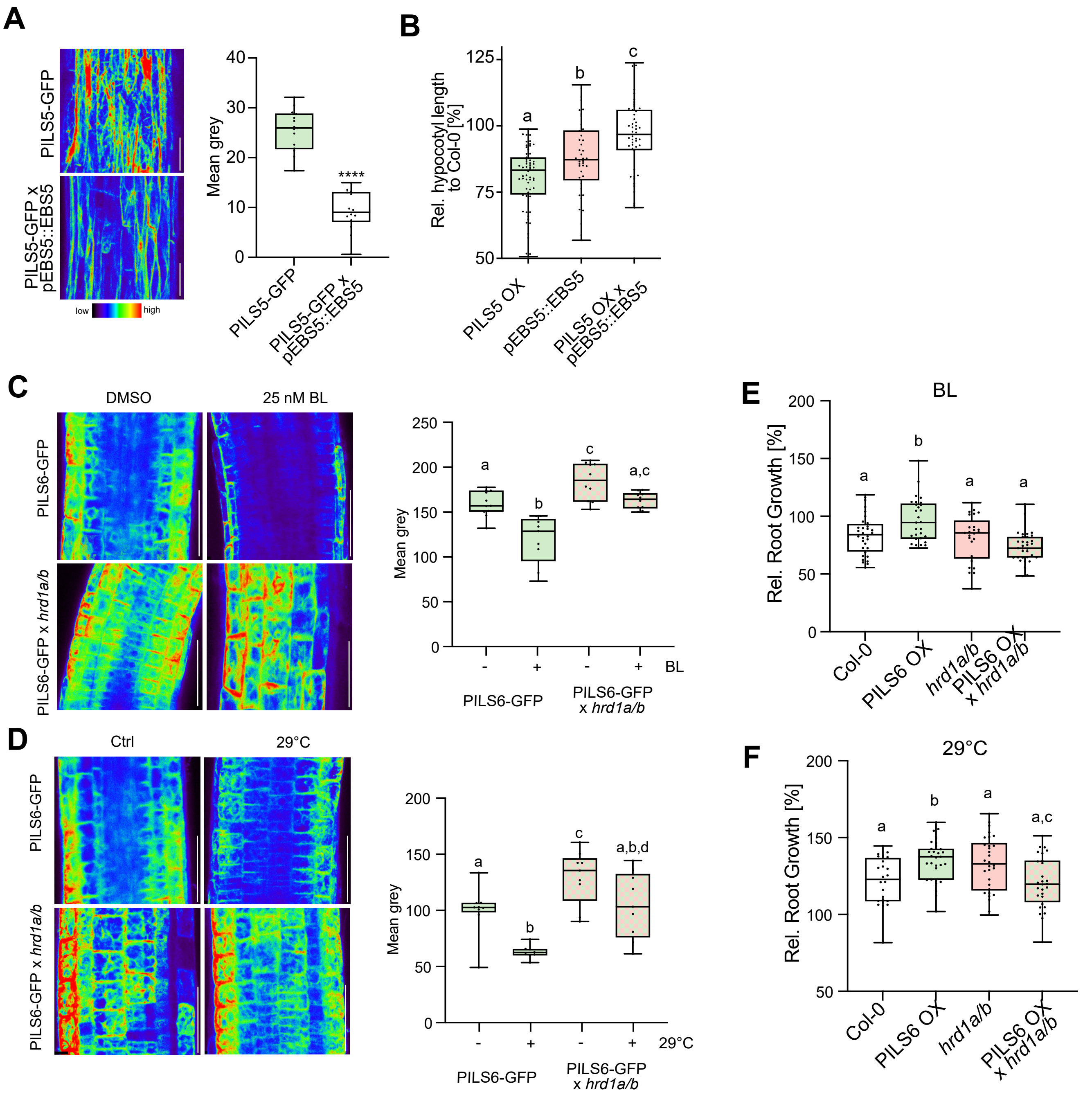

figure S4: HRD1 integrates internal and external signals with PILS turnover.

A, Representative images and quantifications of PILS5-GFP and PILS5-GFP x pEBS5::EBS5 signal in 3-days-old dark-grown seedlings. Scale bars, 50 µm. n = 14, Student's t-test (****P < 0.0001). **B,** Relative hypocotyl length of 3-day-old dark-grown seedlings. n = 45-60. one-way ANOVA followed by Tukey's multiple comparison test (b: pEBS5::EBS5 vs. Col-0 P < 0.05, pEBS5::EBS5 vs. PILS5 OX x pEBS5::EBS5 P < 0.01; c: PILS5 OX x pEBS5::EBS5 vs. PILS5 OX P < 0.001). **C, D,** Representative images and quantifications of PILS6-GFP and PILS6-GFP x *hrd1a hrd1b* signal in roots. 3-days-old seedlings were treated (C) for 2h in liquid ½ MS with DMSO or 25 nM 24-Eppibrassinolide (BL) or **(D)** plates were transferred for 72h to 29°C or kept as control at 21°C. Scale bars, 50 µm. n = 9-11, two-way ANOVA followed by Tukey's multiple comparison test (**A,** b: PILS6-GFP ctrl. vs. BL and PILS6-GFP BL vs. PILS6-GFP x *hrd1a hrd1b* ctrl. P < 0.001, PILS6-GFP BL vs. PILS6-GFP x *hrd1a hrd1b* BL. P < 0.0001; c: PILS6-GFP ctrl. vs. PILS6-GFP x *hrd1a hrd1b* ctrl. P < 0.05; B, b: PILS6-GFP ctrl. vs. 29°C and PILS6-GFP 29°C vs. PILS6-GFP x *hrd1a hrd1b* 29°C. P < 0.01, PILS6-GFP 29°C vs. PILS6-GFP x *hrd1a hrd1b* ctrl. P < 0.001; c: PILS6-GFP ctrl. vs. PILS6-GFP x *hrd1a hrd1b* ctrl. P < 0.05. **E, F,** Relative root length of 3-day-old seedlings which were transferred for 2d to ½ MS media containing DMSO or 100 nM BL **(E)** or 4-day-seedlings which were transferred for 3d to 29°C or kept at 21°C (F). n = 25-33, two-way ANOVA followed by Tukey's multiple comparison test (*E,* b: Col-0 vs. PILS6 OX P < 0.05; PILS6 OX vs. *hrd1a hrd1b* P < 0.01; PILS6 OX vs. PILS6 OX x *hrd1a hrd1b* P < 0.001; **F,** b: Col-0 vs. PILS6 OX P < 0.05; PILS6 OX vs. *hrd1a hrd1b* P < 0.01; c: hrd1a hrd1b vs. PILS6 OX x *hrd1a hrd1b* P < 0.05). In all panels with boxplots: Box limits represent the 25th percentile and 75th percentile; the horizontal line represents the median. Whiskers display min. to max. values. All experiments were repeated at least three times.

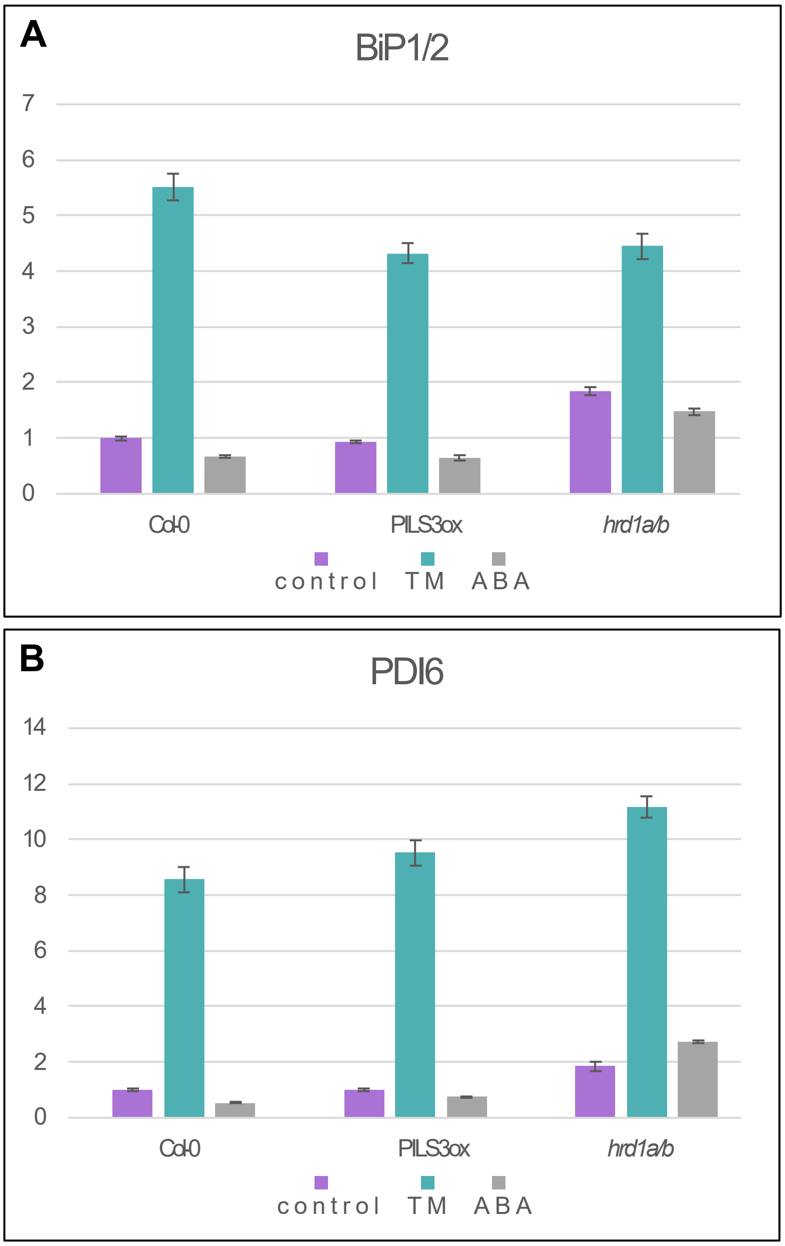

figure S5: ABA treatment does not induce ER stress.
qPCR analysis detecting transcript levels of *BIP1, BIP2*, **(A)** and *PDI6* **(B)** normalized against *UBQ5* and *EIF4*. 4-day-old seedlings were treated for 4 hours with DMSO or 100 nM ABA or 5 µg/ml TM. Bars represent means ± SD, n = 3.

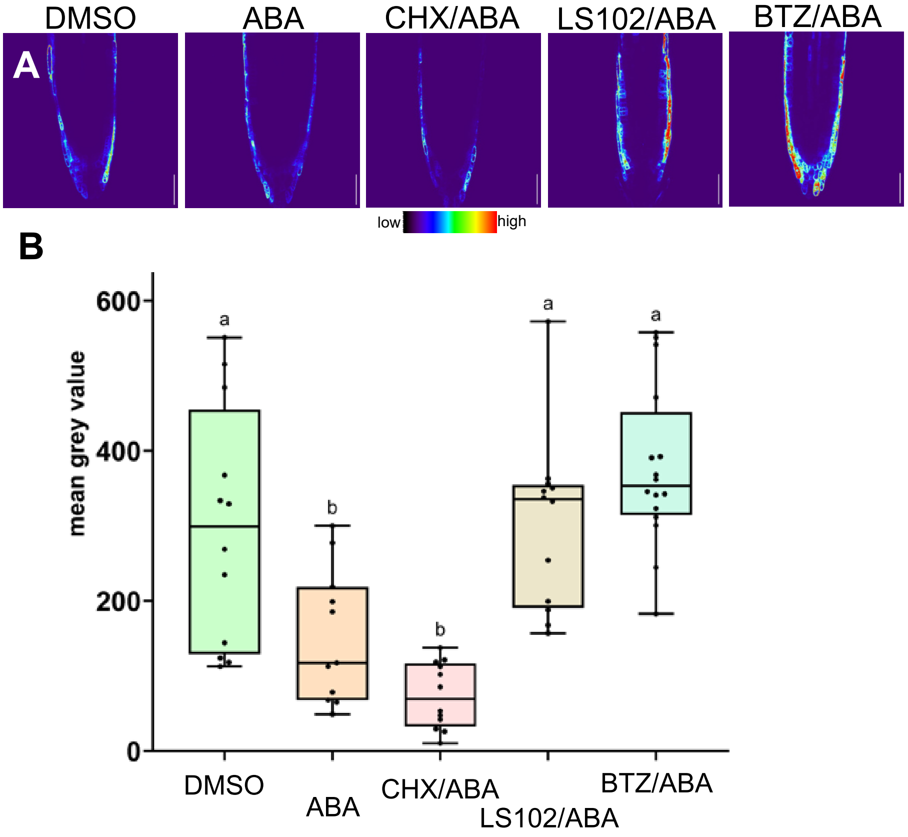

figure S6: Proteasome and HRD1 activity is required for the ABA-induced degradation of PILS3.
A-B, Representative images **(A)** and quantifications **(B)** of GFP-PILS3, signal in 4-day seedlings. Seedlings were grown on solid ½ MS and treated with DMSO and 100 nM ABA as well as with ABA in combination with either 100 µM Cyclohecamide (CHX), 10 µM LS102, or 25 µM BTZ, in liquid ½ MS for 4h. Scale bars, 50 µm. n = 9-15 from seedling replicates pooled, one-way ANOVA followed by Tukey's multiple comparisons between treatments. In all panels with boxplots: Box limits represent 25th percentile and 75th percentile; horizontal line represents median. Whiskers display min. to max. values. P-Values: * $P < 0.05$, ** $P < 0.01$, *** $P < 0.001$, **** $P < 0.0001$. All experiments were repeated at least three times.

Legends for Tables S1, S2 and S3

- **Table S1 (separate xlsx file)**
  Mass Spectrometry identification using GFP-PILS2, GFP-PILS3, and PILS6-GFP as baits.
- **Table S2 (separate xlsx file)**
  Primer List
- **Table S3 (separate xlsx file)**
  Mass spectrometry identification of Hrd1-interacting proteins in Arabidopsis

REFERENCES AND NOTES

1. E. Barbez, M. Kubeš, J. Rolčík, C. Béziat, A. Pěnčík, B. Wang, M. R. Rosquete, J. Zhu, P. I. Dobrev, Y. Lee, E. Zažímalovà, J. Petrášek, M. Geisler, J. Friml, J. Kleine-Vehn, A novel putative auxin carrier family regulates intracellular auxin homeostasis in plants. *Nature* **485**, 119–122 (2012).

2. C. Béziat, E. Barbez, M. I. Feraru, D. Lucyshyn, J. Kleine-Vehn, Light triggers PILS-dependent reduction in nuclear auxin signalling for growth transition. *Nat Plants* **3**, 17105 (2017).

3. E. Feraru, M. I. Feraru, E. Barbez, S. Waidmann, L. Sun, A. Gaidora, J. Kleine-Vehn, PILS6 is a temperature-sensitive regulator of nuclear auxin input and organ growth in Arabidopsis thaliana. *Proc. Natl. Acad. Sci. U.S.A.* **116**, 3893–3898 (2019).

4. J. Mravec, P. Skůpa, A. Bailly, K. Hoyerová, P. Krecek, A. Bielach, J. Petrásek, J. Zhang, V. Gaykova, Y.-D. Stierhof, P. I. Dobrev, K. Schwarzerová, J. Rolcík, D. Seifertová, C. Luschnig, E. Benková, E. Zazímalová, M. Geisler, J. Friml, Subcellular homeostasis of phytohormone auxin is mediated by the ER-localized PIN5 transporter. *Nature* **459**, 1136–1140 (2009).

5. L. Sun, E. Feraru, M. I. Feraru, S. Waidmann, W. Wang, G. Passaia, Z.-Y. Wang, K. Wabnik, J. Kleine-Vehn, PIN-LIKES coordinate brassinosteroid signaling with nuclear auxin input in *Arabidopsis thaliana*. *Curr. Biol.* **30**, 1579–1588.e6 (2020).

6. S. Waidmann, C. Béziat, J. Ferreira Da Silva Santos, E. Feraru, M. I. Feraru, L. Sun, S. Noura, Y. Boutté, J. Kleine-Vehn, Endoplasmic reticulum stress controls PIN-LIKES abundance and thereby growth adaptation. *Proc. Natl. Acad. Sci. U.S.A.* **120**, e2218865120 (2023).

7. E. Feraru, M. I. Feraru, J. Moulinier-Anzola, M. Schwihla, J. Ferreira Da Silva Santos, L. Sun, S. Waidmann, B. Korbei, J. Kleine-Vehn, PILS proteins provide a homeostatic feedback on auxin signaling output. *Development* **149**, dev200929 (2022).

8. R. Strasser, Protein quality control in the endoplasmic reticulum of plants. *Annu. Rev. Plant Biol.* **69**, 147–172 (2018).

9. J. Pollier, T. Moses, M. González-Guzmán, N. De Geyter, S. Lippens, R. Vanden Bossche, P. Marhavý, A. Kremer, K. Morreel, C. J. Guérin, A. Tava, W. Oleszek, J. M. Thevelein, N. Campos, S. Goormachtig, A. Goossens, The protein quality control system manages plant defence compound synthesis. *Nature* **504**, 148–152 (2013).

10. R. D. Etherington, M. Bailey, J.-B. Boyer, L. Armbruster, X. Cao, J. C. Coates, T. Meinnel, M. Wirtz, C. Giglione, D. J. Gibbs, Nt-acetylation-independent turnover of SQUALENE EPOXIDASE 1 by *Arabidopsis* DOA10-like E3 ligases. *Plant Physiol.* **193**, 2086–2104 (2023).

11. Q. Chen, R. Liu, Y. Wu, S. Wei, Q. Wang, Y. Zheng, R. Xia, X. Shang, F. Yu, X. Yang, L. Liu, X. Huang, Y. Wang, Q. Xie, ERAD-related E2 and E3 enzymes modulate the drought response by regulating the stability of PIP2 aquaporins. *Plant Cell* **33**, 2883–2898 (2021).

12. V. G. Doblas, V. Amorim-Silva, D. Posé, A. Rosado, A. Esteban, M. Arró, H. Azevedo, A. Bombarely, O. Borsani, V. Valpuesta, A. Ferrer, R. M. Tavares, M. A. Botella, The *SUD1* gene encodes a putative E3 ubiquitin ligase and is a positive regulator of 3-hydroxy-3-methylglutaryl coenzyme a reductase activity in *Arabidopsis*. *Plant Cell* **25**, 728–743 (2013).

13. P. Baster, S. Robert, J. Kleine-Vehn, S. Vanneste, U. Kania, W. Grunewald, B. De Rybel, T. Beeckman, J. Friml, SCF(TIR1/AFB)-auxin signalling regulates PIN vacuolar trafficking and auxin fluxes during root gravitropism. *EMBO J.* **32**, 260–274 (2013).

14. J. Kleine-Vehn, J. Leitner, M. Zwiewka, M. Sauer, L. Abas, C. Luschnig, J. Friml, Differential degradation of PIN2 auxin efflux carrier by retromer-dependent vacuolar targeting. *Proc. Natl. Acad. Sci. U.S.A.* **105**, 17812–17817 (2008).

15. J. Leitner, K. Retzer, B. Korbei, C. Luschnig, Dynamics in PIN2 auxin carrier ubiquitylation in gravity-responding Arabidopsis roots. *Plant Signal. Behav.* **7**, 1271–1273 (2012).

16. S. Niemes, M. Labs, D. Scheuring, F. Krueger, M. Langhans, B. Jesenofsky, D. G. Robinson, P. Pimpl, Sorting of plant vacuolar proteins is initiated in the ER. *Plant J.* **62**, 601–614 (2010).

17. J. Kleine-Vehn, P. Dhonukshe, M. Sauer, P. B. Brewer, J. Wiśniewska, T. Paciorek, E. Benková, J. Friml, ARF GEF-dependent transcytosis and polar delivery of PIN auxin carriers in Arabidopsis. *Curr. Biol.* **18**, 526–531 (2008).

18. K. Dünser, M. Schöller, A.-K. Rößling, C. Löfke, N. Xiao, B. Pařízková, S. Melnik, M. Rodriguez-Franco, E. Stöger, O. Novák, J. Kleine-Vehn, Endocytic trafficking promotes vacuolar enlargements for fast cell expansion rates in plants. *eLife* **11**, e75945 (2022).

19. A. F. Kisselev, W. A. van der Linden, H. S. Overkleeft, Proteasome inhibitors: An expanding army attacking a unique target. *Chem. Biol.* **19**, 99–115 (2012).

20. S. Waidmann, L. De-Araujo, J. Kleine-Vehn, B. Korbei, Immunoprecipitation of membrane proteins from *Arabidopsis thaliana* root tissue. *Methods Mol. Biol.* **1761**, 209–220 (2018).

21. C. Grefen, P. Obrdlik, K. Harter, The determination of protein-protein interactions by the mating-based split-ubiquitin system (mbSUS). *Methods Mol. Biol.* **479**, 217–233 (2009).

22. D. G. Mehlhorn, N. Wallmeroth, K. W. Berendzen, C. Grefen, 2in1 vectors improve in planta BiFC and FRET analyses. *Methods Mol. Biol.* **1691**, 139–158 (2018).

23. Z. Hong, H. Jin, T. Tzfira, J. Li, Multiple mechanism-mediated retention of a defective brassinosteroid receptor in the endoplasmic reticulum of Arabidopsis. *Plant Cell* **20**, 3418–3429 (2008).

24. W. Su, Y. Liu, Y. Xia, Z. Hong, J. Li, Conserved endoplasmic reticulum-associated degradation system to eliminate mutated receptor-like kinases in Arabidopsis. *Proc. Natl. Acad. Sci. U.S.A.* **108**, 870–875 (2011).

25. Y. Jo, R. A. DeBose-Boyd, Post-translational regulation of HMG CoA reductase. *Cold Spring Harb. Perspect. Biol.* **14**, a041253 (2022).

26. T. Guo, H. Weber, M. C. E. Niemann, L. Theisl, G. Leonte, O. Novák, T. Werner, Arabidopsis HIPP proteins regulate endoplasmic reticulum-associated degradation of CKX proteins and cytokinin responses. *Mol. Plant* **14**, 1918–1934 (2021).

27. J. Li, B. Zhang, P. Duan, L. Yan, H. Yu, L. Zhang, N. Li, L. Zheng, T. Chai, R. Xu, Y. Li, An endoplasmic reticulum-associated degradation–related E2–E3 enzyme pair controls grain size and weight through the brassinosteroid signaling pathway in rice. *Plant Cell* **35**, 1076–1091 (2023).

28. S. Tang, Z. Zhao, X. Liu, Y. Sui, D. Zhang, H. Zhi, Y. Gao, H. Zhang, L. Zhang, Y. Wang, M. Zhao, D. Li, K. Wang, Q. He, R. Zhang, W. Zhang, G. Jia, W. Tang, X. Ye, C. Wu, X. Diao, An E2-E3 pair contributes to seed size control in grain crops. *Nat. Commun.* **14**, 3091 (2023).

29. G. Langin, M. Raffeiner, D. Biermann, M. Franz-Wachtel, D. Spinti, F. Börnke, B. Macek, S. Üstün, ER-anchored protein sorting controls the fate of two proteasome activators for intracellular organelle communication during proteotoxic stress. bioRxiv 571118 [Preprint] (2023). https://doi.org/10.1101/2023.12.11.571118.

30. N. Yagishita, S. Aratani, C. Leach, T. Amano, Y. Yamano, K. Nakatani, K. Nishioka, T. Nakajima, RING-finger type E3 ubiquitin ligase inhibitors as novel candidates for the treatment of rheumatoid arthritis. *Int. J. Mol. Med.* **30**, 1281–1286 (2012).

31. N. Vashistha, S. E. Neal, A. Singh, S. M. Carroll, R. Y. Hampton, Direct and essential function for Hrd3 in ER-associated degradation. *Proc. Natl. Acad. Sci. U.S.A.* **113**, 5934–5939 (2016).

32. P. Che, J. D. Bussell, W. Zhou, G. M. Estavillo, B. J. Pogson, S. M. Smith, Signaling from the endoplasmic reticulum activates brassinosteroid signaling and promotes acclimation to stress in Arabidopsis. *Sci. Signal.* **3**, ra69 (2010).

33. E. M. Neill, M. C. R. Byrd, T. Billman, F. Brandizzi, A. E. Stapleton, Plant growth regulators interact with elevated temperature to alter heat stress signaling via the Unfolded Protein Response in maize. *Sci. Rep.* **9**, 10392 (2019).

34. J. Schoberer, U. Vavra, Y.-J. Shin, C. Grünwald-Gruber, R. Strasser, Elucidation of the late steps in the glycan-dependent ERAD of soluble misfolded glycoproteins. *Plant J.* **121**, e17185 (2025).

35. K. Wu, S. Itskanov, D. L. Lynch, Y. Chen, A. Turner, J. C. Gumbart, E. Park, Substrate recognition mechanism of the endoplasmic reticulum-associated ubiquitin ligase Doa10. *Nat. Commun.* **15**, 2182 (2024).

36. T. Noguchi, S. Fujioka, S. Choe, S. Takatsuto, S. Yoshida, H. Yuan, K. A. Feldmann, F. E. Tax, Brassinosteroid-insensitive dwarf mutants of Arabidopsis accumulate brassinosteroids. *Plant Physiol.* **121**, 743–752 (1999).

37. C. Grefen, S. Lalonde, P. Obrdlik, Split-ubiquitin system for identifying protein-protein interactions in membrane and full-length proteins. *Curr. Protoc. Neurosci.* **41**, 5.27.1–5.27.41 (2007).

38. C. Grefen, M. R. Blatt, A 2in1 cloning system enables ratiometric bimolecular fluorescence complementation (rBiFC). *Biotechniques* **53**, 311–314 (2012).

39. A. Hecker, N. Wallmeroth, S. Peter, M. R. Blatt, K. Harter, C. Grefen, Binary 2in1 vectors improve in planta (Co)localization and dynamic protein interaction studies. *Plant Physiol.* **168**, 776–787 (2015).

40. A. Castilho, Ed., *Glyco-Engineering: Methods and Protocols*, vol. **1321** of *Methods in Molecular Biology* (Springer New York, 2015).

41. J. Rappsilber, M. Mann, Y. Ishihama, Protocol for micro-purification, enrichment, pre-fractionation and storage of peptides for proteomics using StageTips. *Nat. Protoc.* **2**, 1896–1906 (2007).

42. S. Tyanova, T. Temu, J. Cox, The MaxQuant computational platform for mass spectrometry-based shotgun proteomics. *Nat. Protoc.* **11**, 2301–2319 (2016).

43. R. D. Gietz, R. A. Woods, Transformation of yeast by lithium acetate/single-stranded carrier DNA/polyethylene glycol method. *Methods Enzymol.* **350**, 87–96 (2002).

44. S. N. W. Hoernstein, B. Özdemir, N. Van Gessel, A. A. Miniera, B. Rogalla Von Bieberstein, L. Nilges, J. Schweikert Farinha, R. Komoll, S. Glauz, T. Weckerle, F. Scherzinger, M. Rodriguez-

Franco, S. J. Müller-Schüssele, R. Reski, A deeply conserved protease, acylamino acid-releasing enzyme (AARE), acts in ageing in Physcomitrella and Arabidopsis. *Commun. Biol.* **6**, 61 (2023).

45. C. S. Hughes, S. Moggridge, T. Müller, P. H. Sorensen, G. B. Morin, J. Krijgsveld, Single-pot, solid-phase-enhanced sample preparation for proteomics experiments. *Nat. Protoc.* **14**, 68–85 (2019).

46. M. C. Chambers, B. Maclean, R. Burke, D. Amodei, D. L. Ruderman, S. Neumann, L. Gatto, B. Fischer, B. Pratt, J. Egertson, K. Hoff, D. Kessner, N. Tasman, N. Shulman, B. Frewen, T. A. Baker, M.-Y. Brusniak, C. Paulse, D. Creasy, L. Flashner, K. Kani, C. Moulding, S. L. Seymour, L. M. Nuwaysir, B. Lefebvre, F. Kuhlmann, J. Roark, P. Rainer, S. Detlev, T. Hemenway, A. Huhmer, J. Langridge, B. Connolly, T. Chadick, K. Holly, J. Eckels, E. W. Deutsch, R. L. Moritz, J. E. Katz, D. B. Agus, M. MacCoss, D. L. Tabb, P. Mallick, A cross-platform toolkit for mass spectrometry and proteomics. *Nat. Biotechnol.* **30**, 918–920 (2012).

47. J. Cox, M. Y. Hein, C. A. Luber, I. Paron, N. Nagaraj, M. Mann, Accurate proteome-wide label-free quantification by delayed normalization and maximal peptide ratio extraction, termed MaxLFQ. *Mol. Cell. Proteomics* **13**, 2513–2526 (2014).

48. C. Cheng, V. Krishnakumar, A. P. Chan, F. Thibaud-Nissen, S. Schobel, C. D. Town, Araport11: A complete reannotation of the *Arabidopsis thaliana* reference genome. *Plant J.* **89**, 789–804 (2017).

49. L. Käll, J. D. Canterbury, J. Weston, W. S. Noble, M. J. MacCoss, Semi-supervised learning for peptide identification from shotgun proteomics datasets. *Nat. Methods* **4**, 923–925 (2007).

50. A. I. Nesvizhskii, A. Keller, E. Kolker, R. Aebersold, A statistical model for identifying proteins by tandem mass spectrometry. *Anal. Chem.* **75**, 4646–4658 (2003).

51. V. Demichev, C. B. Messner, S. I. Vernardis, K. S. Lilley, M. Ralser, DIA-NN: Neural networks and interference correction enable deep proteome coverage in high throughput. *Nat. Methods* **17**, 41–44 (2020).

52. M. E. Ritchie, B. Phipson, D. Wu, Y. Hu, C. W. Law, W. Shi, G. K. Smyth, limma powers differential expression analyses for RNA-sequencing and microarray studies. *Nucleic Acids Res.* **43**, e47 (2015).

53. Y. Benjamini, Y. Hochberg, Controlling the false discovery rate: A practical and powerful approach to multiple testing. *J. R. Stat. Soc. Series B Stat. Methodol.* **57**, 289–300 (1995).

54. G. Yu, L.-G. Wang, Y. Han, Q.-Y. He, clusterProfiler: An R package for comparing biological themes among gene clusters. *OMICS* **16**, 284–287 (2012).
